# Homogeneous tumor targeting with a single dose of HER2-targeted albumin-binding domain-fused nanobody-drug conjugates results in long-lasting tumor remission in mice

**DOI:** 10.7150/thno.57510

**Published:** 2021-03-13

**Authors:** Katerina T Xenaki, Bram Dorresteijn, Joey A Muns, Kevin Adamzek, Sofia Doulkeridou, HendrikJan Houthoff, Sabrina Oliveira, Paul MP van Bergen en Henegouwen

**Affiliations:** 1 Cell Biology, Neurobiology and Biophysics, Department of Biology, Faculty of Science, Utrecht University, Utrecht, The Netherlands.; 2 LinXis B.V., Amsterdam, The Netherlands.; 3 Pharmaceutics, Department of Pharmaceutical Sciences, Faculty of Science, Utrecht University, Utrecht, The Netherlands.

**Keywords:** nanobody, single-domain antibodies, albumin-binding domain, half-life extension, nanobody-drug conjugates

## Abstract

**Background:** The non-homogenous distribution of antibody-drug conjugates (ADCs) within solid tumors is a major limiting factor for their wide clinical application. Nanobodies have been shown to rapidly penetrate into xenografts, achieving more homogeneous tumor targeting. However, their rapid renal clearance can hamper their application as nanobody drug conjugates (NDCs). Here, we evaluate whether half-life extension via non-covalent interaction with albumin can benefit the efficacy of a HER2-targeted NDC.

**Methods:** HER2-targeted nanobody 11A4 and the irrelevant nanobody R2 were genetically fused to an albumin-binding domain (ABD) at their C-terminus. Binding to both albumin and tumor cells was determined by ELISA-based assays. The internalization potential as well as the *in vitro* efficacy of NDCs were tested on HER2 expressing cells. Serum half-life of iodinated R2 and R2-ABD was studied in tumor-free mice. The distribution of fluorescently labelled 11A4 and 11A4-ABD was assessed *in vitro* in 3D spheroids. Subsequently, the *in vivo* distribution was evaluated by optical molecular imaging and *ex vivo* by tissue biodistribution and tumor immunohistochemical analysis after intravenous injection of IRDye800-conjugated nanobodies in mice bearing HER2-positive subcutaneous xenografts. Finally, efficacy studies were performed in HER2-positive NCI-N87 xenograft-bearing mice intravenously injected with a single dose (250 nmol/kg) of nanobodies conjugated to auristatin F (AF) either via a maleimide or the organic Pt(II)‑based linker, coined L*x*^®^.

**Results:** 11A4-ABD was able to bind albumin and HER2 and was internalized by HER2 expressing cells, irrespective of albumin presence. Interaction with albumin did not alter its distribution through 3D spheroids. Fusion to ABD resulted in a 14.8-fold increase in the serum half-life, as illustrated with the irrelevant nanobody. Furthermore, ABD fusion prolonged the accumulation of 11A4-ABD in HER2-expressing xenografts without affecting the expected homogenous intratumoral distribution. Next to that, reduced kidney retention of ABD-fused nanobodies was observed. Finally, a single dose administration of either 11A4-ABD-maleimide-AF or 11A4-ABD-*Lx*-AF led to long-lasting tumor remission in HER2-positive NCI-N87 xenograft-bearing mice.

**Conclusion:** Our results demonstrate that genetic fusion of a nanobody to ABD can significantly extend serum half-life, resulting in prolonged and homogenous tumor accumulation. Most importantly, as supported by the impressive anti-tumor efficacy observed after a single dose administration of 11A4-ABD-AF, our data reveal that monovalent internalizing ABD-fused nanobodies have potential for the development of highly effective NDCs.

## Introduction

The rapidly expanding field of antibody drug conjugates (ADCs) utilizes the high antigen binding specificity of monoclonal antibodies (mAbs) to selectively deliver a covalently linked chemotherapeutic drug to tumors 1. This cancer cell-specific delivery of the linked cytotoxic drug allows for lower minimum effective doses. Furthermore, the systemic toxicity such a drug could cause if administered alone is alleviated, enabling for higher maximum tolerated doses (MTD) and overall wider therapeutic windows 2. Several aspects regarding the design of ADCs can influence their antitumor efficacy. Besides the choice of antigen target for the mAb part of an ADC 3, the design of linkers and the optimization of conjugation strategies, as well as the available cytotoxic drugs are critical considerations being greatly explored, especially for ADCs targeting solid tumors 2.

To date, two out of the nine clinically approved ADCs are used for the treatment of HER2 expressing solid tumors 4. Both, Kadcyla^®^ (Trastuzumab emtansine or T-DM1) and Enhertu^®^ (Trastuzumab deruxtecan) consist of the humanized HER2-targeted mAb trastuzumab, while employing two different types of linkers, *i.e.* a non-cleavable linker for Kadcyla and an enzymatically cleavable linker for Enhertu 5,6. Next to the different linker technologies, the drug payloads used are displaying distinct modes of action. DM1 used in Kadcyla is a tubulin polymerization inhibitor, like the majority of drug payloads used in ADC clinical trials 4. It has been shown that after lysosomal degradation, the released Lys-SMCC-DM1 cannot passively diffuse through the plasma membrane to the tumor microenvironment 7, a common characteristic of hydrophilic drug payloads that are therefore unable to kill neighboring cells through a bystander mechanism 8. On the other hand, drugs, such as Enhertu's topoisomerase I inhibitor deruxtecan, demonstrating high membrane permeability could result in cytotoxicity against neighboring (tumor) cells that are not directly targeted by the ADC, conferring bystander cell killing effect. However, the same mechanism might also contribute to decreased tolerability and lower MTD. Differences in the *in vivo* efficacy and tolerability of these HER2-targeted ADCs can therefore be attributed to different payloads as well as to different linker types used.

Another major aspect greatly affecting the efficacy of ADCs is their heterogeneous intra-tumoral distribution 9. Besides tumor microenvironment characteristics 10, vascularization 11, antigen expression 12 and clearance rates 13, the physicochemical mAb characteristics, like size and high binding affinities 14,15, can also result in slow tumor penetration. Often, mAb distribution is restricted to a few cell layers surrounding blood vessels (resulting in the 'binding site-barrier effect') and it is generally poor at hypoxic areas of tumors, resulting in suboptimal drug delivery 16-18. Intra-tumoral distribution is also mAb dose dependent, meaning that ADCs to be used at a low MTD will be prone to heterogenous distribution. Especially for ADCs conjugated to non-bystander drugs and/or ADCs that have to be used at low MTD, improvements in intra-tumoral distribution are important for increasing their *in vivo* efficacy 19. Ideally, what needs to be achieved is the delivery of a certain amount of payload per cell throughout the tumor, sufficient for killing each individual cell 20,21.

Antibody fragments of smaller molecular weight, resulting in higher diffusivity 14,22 and vascular permeability 23, have been shown to demonstrate more homogeneous tissue distribution over mAbs 14,24-26. Over the past years, especially after the FDA approval of caplacizumab 27, nanobodies (registered trademark of Ablynx), also known as single-domain antibodies (sdAb), have been receiving growing attention 28,29. Nanobodies constitute the antigen-binding domain of the heavy-chain only antibodies first identified in members of the Camelidae family almost 30 years ago 30. In contrast to other small (artificial) antibody fragments, nanobodies have the advantage of being derived from naturally binding affinity-matured libraries following immunization and they demonstrate excellent solubility and thermostability 31. Conjugation of nanobodies to radio-isotopes or fluorescent dyes has already led to promising diagnostic imaging and tumor therapeutic applications in pre-clinical, but also clinical set-ups 25,31-35.

A major limitation arising when nanobodies are intended to be used as cytotoxic drug carriers in the form of nanobody-drug conjugates (NDCs) is their short presence in the circulation, as a result of fast renal clearance. The use of albumin and albumin-interacting moieties, due to the direct size increase above the renal filtration threshold (~40-50 kDa, 2-6 nm) and because it allows for neonatal Fc receptor (FcRn)-mediated recycling, results in *in vivo* half-life extension of small biotherapeutics and has been widely used in the clinic 36,37. To minimally affect the exceptional tissue distribution of nanobodies, non-covalent interaction with albumin, by fusion to albumin binding peptides 36 or albumin binding domains (ABD) 38,39, could be an effective approach to extent their half-life.

Another challenge in NDC development and production is related to solubility issues, taking into account that sometimes hydrophobic drugs have to be coupled to the small-sized hydrophilic nanobodies in a stable and inert way. For this purpose, the cationic metal-organic Pt(II)‑based linker, [ethylenediamineplatinum(II)]^2+^, coined “L*x*”^®^, has shown great promise 40,41. *Lx*‑technology comprises a two-step approach. In the first step (“complexation”), the drug is coupled to the precursor complex L*x*I_2_. These storable “semi‑final” products contain a positively charged Pt(II) center, which increases the water solubility of drug-linker moieties compared to analogous non‑*Lx* containing constructs. In the second step, these drug-linker moieties can be conjugated either to histidine residues of unmodified intact mAbs in a site-restricted way (meaning that conjugation occurs preferentially to the Fc domain) 42, or to cysteine residues 43 that can be engineered at the C-terminus of nanobodies for site-specific coupling.

In the present study, we evaluated the previously characterized HER2-targeted nanobody 11A4 44 genetically fused to an ABD 39,45 as a potential targeting moiety for developing NDCs. When targeting HER2, the rapid recycling of the receptor 46,47 could negatively affect the cytoplasmic concentration of the released drug, influencing NDC efficacy. Despite the fact that receptor clustering using biparatopic antibodies or nanobodies has been shown to increase endocytosis and lysosomal trafficking 7,34,48, comparative *in vivo* imaging studies using mono- as well as multi-valent nanobody formats suggested an advantage for monovalent constructs with respect to distribution and cell targeting 21,49,50. Therefore, the monovalent 11A4-ABD was chosen for coupling the highly potent microtubule stabilizing drug auristatin F (AF). AF was coupled either via common maleimide-thiol conjugation or using the platinum-based *Lx* linker technology 40, to allow the comparison between these conjugation technologies for NDC applications. The distribution of both 11A4 and 11A4-ABD was assessed *in vitro* in 3D spheroids and *in vivo* in a HER2-positive and negative subcutaneous breast cancer model in mice. The *in vivo* efficacy of the NDCs was evaluated in mice bearing xenografts of HER2 overexpressing cells. Our data confirm that half-life extension of monovalent nanobodies can result in increased homogenous tumor accumulation, significantly enhancing the *in vivo* efficacy of the NDC, even after single-dose administration.

## Results

### 11A4-ABD maintains high binding affinity and is internalized even in the presence of albumin

**Figure 1:**
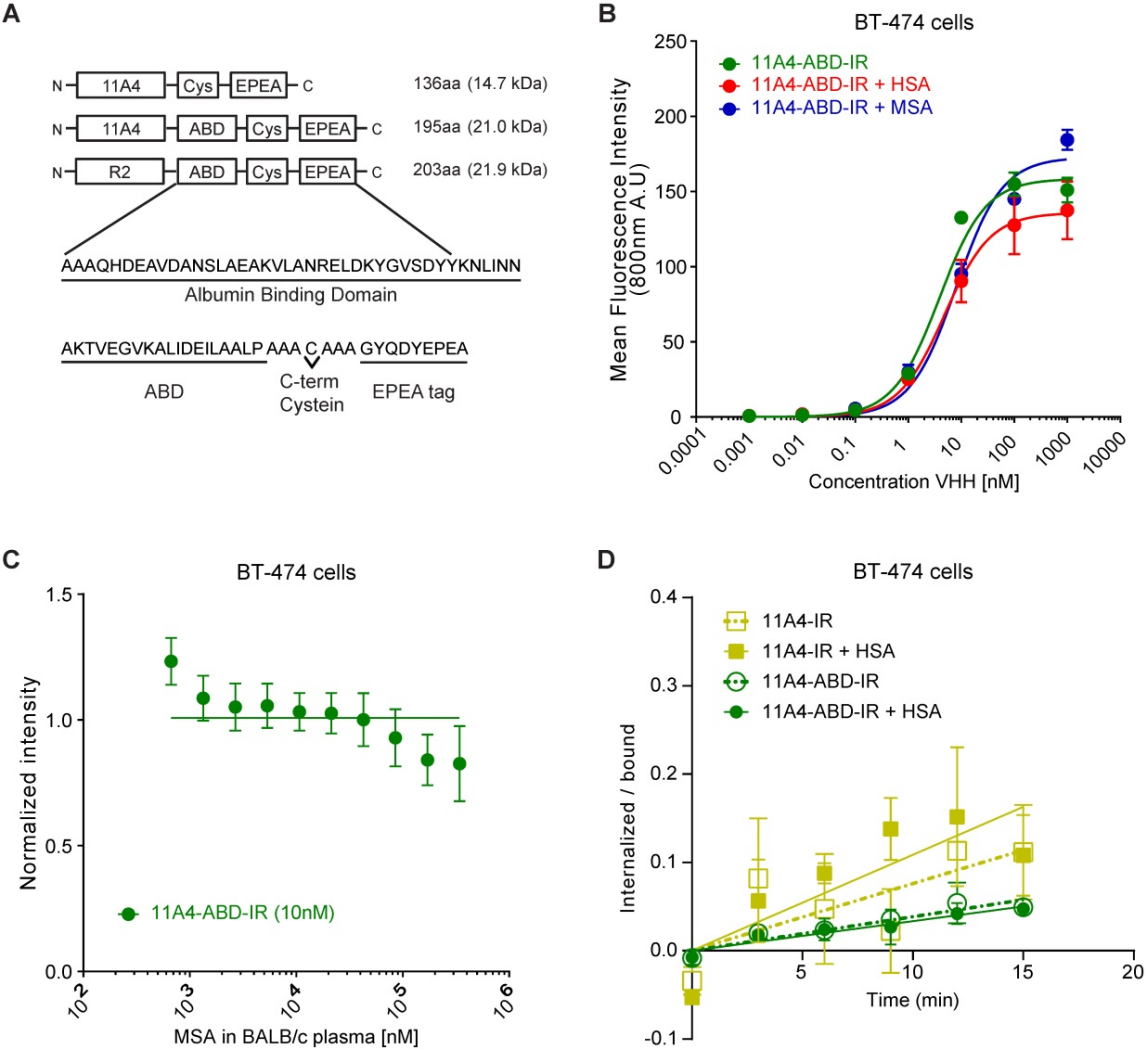
** 11A4-ABD shows high binding affinity and is internalized in HER2 expressing cells, regardless of albumin presence. A)** Schematic representation of the different nanobody constructs. **B)** Binding to HER2 expressing BT-474 cells of IRDye800 conjugated 11A4-ABD (11A4-ABD-IR) alone (green) or in the presense of human serum albumin (HSA; red) or mouse serum albumin (MSA; blue). **C)** Competition binding of 11A4-ABD-IR to BT-474 cells in the presence of BALB/c mouse serum. **D)** Ratio of internalized over bound signal of 11A4-IR (yellow squares) or 11A4-ABD-IR (green circles) in the absence (open symbols, dashed lines) or presense of HSA (filled symboles, continuous lines) over time on BT-474 cells. The data shown are representative experiments. Values were plotted as mean ±SD (n = 3).

For generating a nanobody drug conjugate (NDC), we made use of the nanobody 11A4 that was previously selected in our group to selectively bind HER2 44. In order to achieve extension of serum half-life *in vivo*, 11A4, as well as the irrelevant nanobody R2, were genetically fused at their C-terminus to the sequence encoding for the albumin-binding domain (ABD; G148-GA3) derived from the Streptococcal protein G 39. A C-terminal free cysteine was included in all sequences, to allow for site-specific conjugation. This was followed by a C-tag (also termed EPEA tag) to facilitate protein purification (Figure 1A). The different nanobody constructs were produced in *E. coli* and C-tag purification yielded protein samples of high purity. Functionality of the free cysteine was confirmed, as site-specific conjugation to either maleimide-IRDye800CW (abbreviated IR; Figure S1A) or maleimide-Alexa488 (abbreviated A488) resulted in conjugates with high degrees of conjugation (DoC; 11A4-IR: 0.7-0.8, 11A4-ABD-IR: 0.8-0.9 and R2-ABD-IR: 0.8-1.0. 11A4-A488 and 11A4-ABD-A488: 0.9). It was confirmed that both HER2-targeted nanobody conjugates specifically bind to HER2-over-expressing BT-474 cells, with similar affinities (K_D, 11A4_ = 8.3 ± 2.6 nM, K_D, 11A4-ABD_ = 3.0 ± 0.4 nM; Figure S1B), whereas no binding was observed on HER2-negative MDA-MB-231 cells. No binding of the irrelevant control R2-ABD-IR was detected on either of the cell lines. These findings illustrate that the fusion of the ABD has no influence on either the binding specificity or the affinity of 11A4 (Figure S1B).

The nanobody-ABD fusions showed high binding affinity for both mouse serum (MSA; K_D,MSA_: 5.5 ± 1.6 nM) and human serum albumin (HSA; K_D,MSA_: 6.6 ± 1.8 nM), while no binding to bovine serum albumin (BSA) was observed (Figure S1C), in agreement with previously reported data 45,51. Consequently, BSA could be used as a blocking agent for the subsequent ELISA-based assays. Importantly, pre-incubation of 11A4-ABD with either MSA or HSA, to allow a complex formation with albumin, minimally affected the apparent binding affinity of 11A4-ABD-albumin complex on BT-474 cells (Figure 1B; K_D, +HSA_ = 4.8 ± 1.0 nM, K_D, +MSA_ = 8.0 ± 1.4 nM, compared to K_D, 11A4-ABD_ = 3.0 ± 0.4 nM). Since 11A4-ABD was intended to be used *in vivo*, we evaluated whether physiologically relevant concentrations of albumin could compete with HER2 binding. As indicated by the line fitted for competitive binding, there is no competition observed with either MSA (Figure 1C) or HSA (Figure S1D) for 11A4-ABD-IR binding on BT-474 cells, even when binding was assessed in 100% BALB/c mouse plasma (~0.342 mM MSA) or 100 mg/ml (1.5 mM) HSA.

Nanobody endocytosis, essential for NDC efficacy, was evaluated using BT-474 cells. Both 11A4 and 11A4-ABD were able to internalize during this 15 min incubation (Figure 1D), either in the presence or absence of HSA (k_e,11A4_: 0.0076 ± 0.002 min^-1^, k_e,11A4+HSA_: 0.0108 ± 0.0025 min^-1^, k_e,11A4-ABD_: 0.0038 ± 0.0005 min^-1^, k_e,11A4-ABD+HSA_: 0.0033 ± 0.0005 min^-1^). These data suggested that a monovalent NDC based on these 11A4 constructs would be able to mediate intracellular drug delivery.

### 11A4-ABD maintains the diffusion characteristics of 11A4 *in vitro*

**Figure 2:**
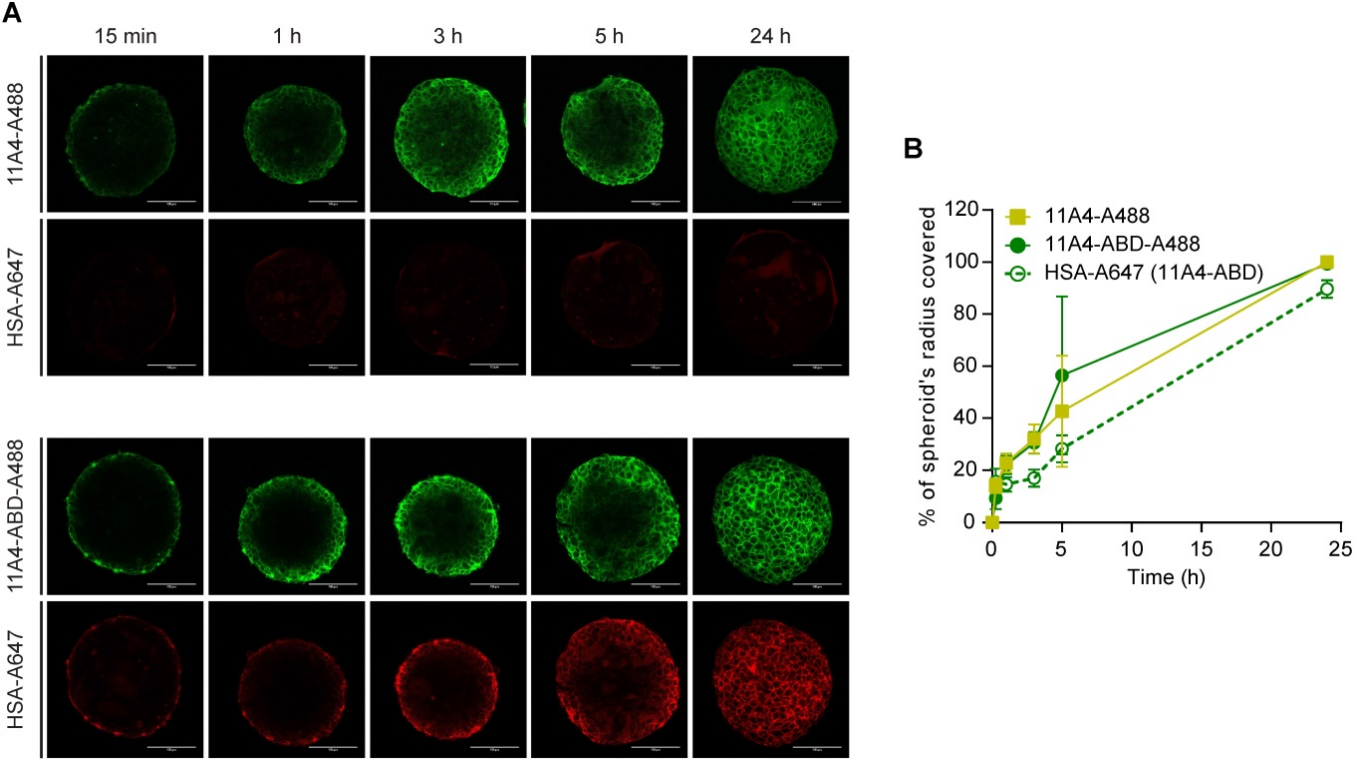
** Distribution of fluorescent nanobodies in HSA pre-treated BT-474 spheroids. A)** Representative confocal images of a central z-plane of BT-474 spheroids incubated with 25 nM of Alexa488 conjugated 11A4 (11A4-A488) or 11A4-ABD (11A4-ABD-A488) in the presence of equimolar amount of Alexa647-conjugated HSA (HSA-A647). Scale bar = 100 µm. **B)** Displacement profile, expressed as % of spheroid's radius, of 11A4-A488 (yellow squares), 11A4-ABD-A488 (green filled circles, continuous line) and HSA-A647 in 11A4-ABD-incubated spheroids (green open circles, dashed line) over time. No HSA-A647 signal was observed in 11A4-A488-incubated spheroids. For quantification details see also Figure S2. Values were plotted as mean ± SD (n = 10) and data points are linked with a connecting line.

To study the diffusion characteristics of 11A4-ABD in the presence of HSA, distribution studies in three-dimensional *in vitro* BT-474 spheroids were performed. Spheroids pre-treated with HSA-A647 were incubated with either 11A4-A488 or 11A4-ABD-A488 in the presence of equimolar amounts of HSA-Alexa647 (HSA-A647) for different time intervals. Fixed samples were imaged and the distribution of the bound fluorescent probes, at a z-plane around the middle of the spheroid, was further analyzed (Figure 2A, S2). Images were thresholded just above background. Based on the percentage of the covered area, the probe's displacement was calculated (Figure S2), expressed as a percentage of spheroid radius and plotted over time (Figure 2B). Both 11A4-A488 and 11A4-ABD-A488 rapidly penetrated into the spheroid core, resulting in coverage of approximately 50% of the spheroid's radius already after 5 h of incubation (Figure 2). HSA-A647 signal was only detected in the presence of 11A4-ABD-A488, following a similar penetration pattern as 11A4-ABD-A488 itself, indicating that the latter construct can simultaneously bind to albumin and HER2 (Figure 2A, B). Despite the presence of HSA, 11A4-ABD-A488 and 11A4-A488 showed a similar trend, with both of them distributing throughout the spheroids within 24 h, suggesting that interaction with HSA via the ABD does not hamper the penetration of 11A4-ABD.

### Fusion to ABD extends the serum half-life of nanobodies

**Figure 3:**
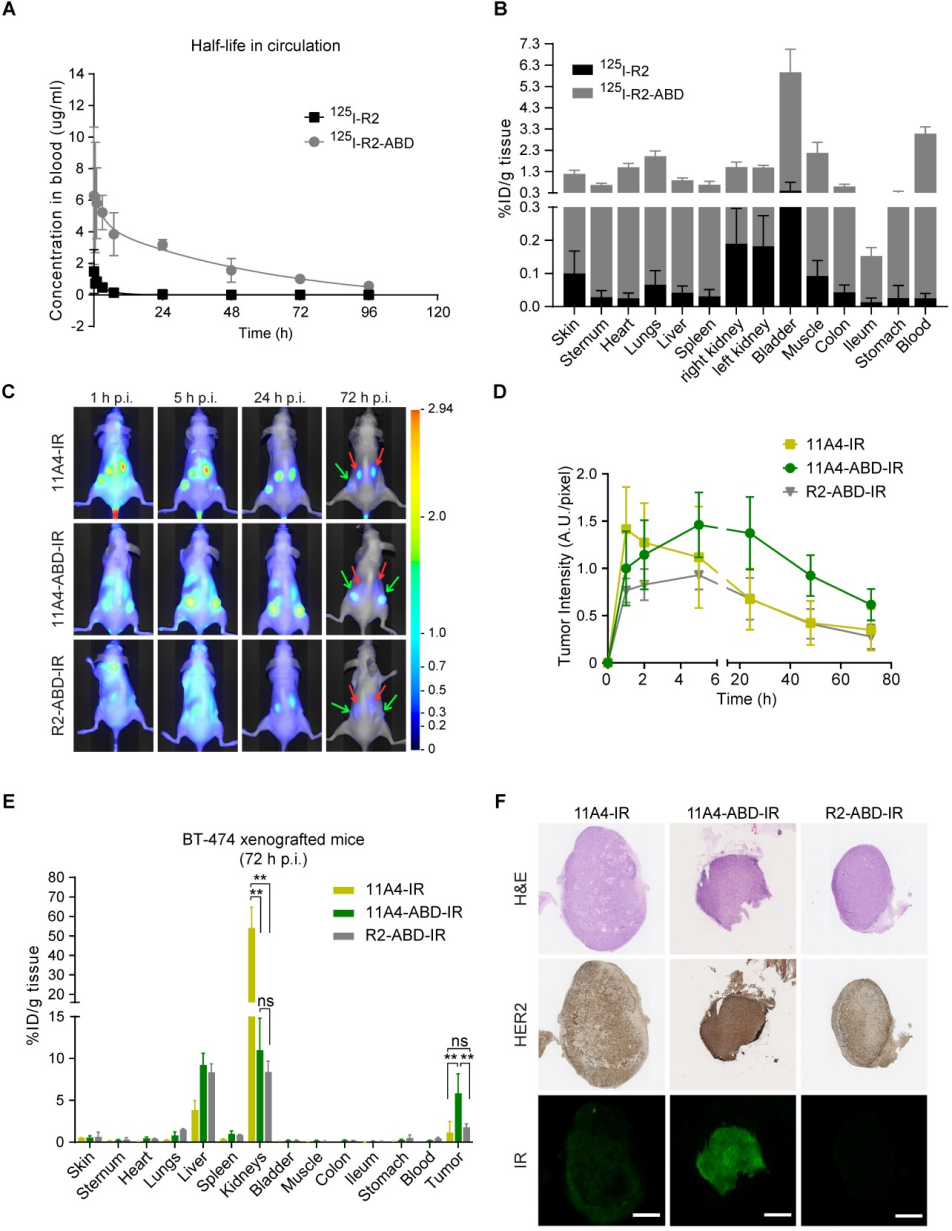
** Nanobody fusion to ABD extends serum half-life and prolongs 11A4-ABD's tumor accumulation. A)** *In vivo* serum pharmacokinetics up to 4 days (mean concentration (µg/mL) ± SD, n = 4) and **B)** Biodistribution at day 4 post injection (p.i.) of ^125^I radiolabeled R2 (black) and R2-ABD (grey) intravenously administered to BALB/c nude mice. **C)** Representative images of BT-474 tumor-bearing mice after single dose administration (50 μg/ mouse) of IR labeled probes. Red arrow: kidney and green arrow: tumors. **D)** Fluorescence intensity at the tumor site over time (mean ± SD); **E)** Biodistribution (mean %ID /g ± SD; ** p<0.002, Welch's t-test) and F) IHC of resected tumors (Scale bar = 1 mm) 72 h p.i. from mice injected with 11A4-IR (yellow, n = 2 mice, 4 tumors), 11A4-ABD-IR (green, n = 4, 8 tumors) or R2-ABD-IR (grey, n = 4, 8 tumors).

To explicitly assess the effect of ABD-fusion on the serum half-life of nanobodies, the irrelevant nanobody R2 was used in order to avoid any pharmacokinetic alterations resulting from a possible target-specific nanobody interaction (“sink effect”). The *in vivo* serum half-life of both iodinated R2 and R2-ABD (SEC-purified; Figure S3) was evaluated in tumor-free mice that were injected with a single dose of ^125^I-probe. After measuring blood radioactivity levels at different time points post injection (p.i.), it was confirmed that fusion to ABD resulted in a 14.8-fold increase in serum half-life (Figure 3A; ^125^I-R2 τ^β^_1/2_: 3.0 h and ^125^I-R2-ABD τ^β^_1/2_: 44.5 h). This increase gave rise to larger areas under the curve (AUC; AUC_R2_: 5.7 μg•h•mL^-1^ and AUC_R2-ABD_: 201.3 μg•h•mL^-1^) that translate to decreased clearance rates (CL_R2_: 2.17 mL•h^-1^ and CL_R2-ABD:_ 0.09 mL•h^-1^) for the irrelevant nanobody R2-ABD, compared to R2. The distribution volumes calculated suggest broad extravascular distribution for both, with R2-ABD being slightly less distributed (V_d, R2_: 9.4 mL and V_d, R2-ABD:_ 5.6 mL). Importantly, no non-specific accumulation, suggesting non-specific targeting via the ABD, was observed for R2-ABD at any of the organs analyzed 4 days after probe administration (Figure 3B). Because of its extended serum half-life, ^125^I-R2-ABD was still detected in the blood of mice 4 days after administration (3.056 ± 0.318 %ID/g tissue), at levels 125 times higher compared to that of ^125^I-R2 (0.024 ± 0.015 %ID/g tissue) (Figure 3B).

### 11A4-ABD shows prolonged tumor accumulation *in vivo*

To evaluate the influence of half-life extension on tumor accumulation, BALB/c nude mice bearing BT-474 tumors were intravenously injected with a single dose of either 11A4-IR, 11A4-ABD-IR or R2-ABD-IR allowing for molecular optical imaging analysis. At early time points, fluorescent signal was observed throughout the entire body of the mice for all three probes (Figure 3C, S4A), as quantified by the background fluorescence measured at a non-tumor skin region of the pelvic area (Figure S4B). For both HER2-targeted nanobodies, tumor-specific accumulation was observed already 1 h p.i. in BT-474 but not in control MDA-MB-231 xenografted mice (Figure 3C-D, S4A, S5A, S5C). Fluorescence signal of 11A4-ABD-IR at the tumor showed a gradual increase, reaching a maximum at 5 h p.i. It remained high for more than 24 h, while 3 days p.i. it was still present at levels above background, demonstrating a prolonged tumor accumulation when compared to 11A4-IR (Figure 3D, S4). In both BT-474 and MDA-MB-231 xenografted mice, background signal levels followed the same trend overtime (Figure S4B, S5B). In line with their longer half-life, background signal of the ABD-IR nanobodies persisted longer than 11A4-IR (Figure S4B, S5B). Instead, due to its rapid renal clearance, as highlighted by the prominent signal in the kidneys, 11A4-IR background signal started fading away already 1 h p.i. Indicative of the different pharmacokinetics of ABD-fused nanobodies was their strongly diminished signal in the kidneys of mice bearing either HER2-positive or negative xenografts (Figure S4A, S5A).

Due to tissue and skin-mediated fluorescence absorption and scattering, as well as positioning of the tumors, optical molecular imaging analysis is considered semi-quantitative. For quantitative analysis of the IR-labeled nanobody uptake in tumors and organs, a biodistribution on tissues resected 72 h p.i. was performed (Figure 3E). In BT-474 tumor bearing mice, 11A4-ABD-IR showed a significantly higher tumor accumulation over 11A4-IR (5.8 ± 2.3 %ID/g tissue for 11A4-ABD-IR and 1.1 ± 1.3 %ID/g tissue for 11A4-IR) and the irrelevant control R2-ABD-IR (1.8 ± 0.4 %ID/g tissue) (Figure 3E). Furthermore, immunohistochemistry of resected BT-474 tumors indicated a homogeneous distribution for 11A4-ABD-IR 72 h p.i. (Figure 3F). The fluorescent signal of 11A4-IR was barely detectable compared to 11A4-ABD-IR, being in agreement with the imaging and biodistribution data. This tumor accumulation was HER2 target-specific, as no probe was retained in MDA-MB-231 xenografts (Figure S5D). Of equal importance was the statistically significant reduction in kidney retention of the ABD-bearing nanobodies when compared to 11A4-IR (54.0 ± 10.7 %ID/g tissue for 11A4-IR, versus 10.9 ± 3.8 % and 8.4 ± 1.3 % ID/g tissue for 11A4-ABD-IR and R2-ABD-IR, respectively). This reduction was accompanied by a significant increase in liver content of ABD-containing probes (11A4-IR: 3.8 ± 1.2 %ID/g tissue, 11A4-ABD-IR: 9.2 ± 1.4 %ID/g tissue, R2-ABD-IR: 8.3 ± 1.0 %ID/g tissue) being in agreement with their altered pharmacokinetics and different clearance routes, as also indicated by the imaging study.

### Both 11A4 and 11A4-ABD auristatin F conjugates are highly potent *in vitro*

**Figure 4:**
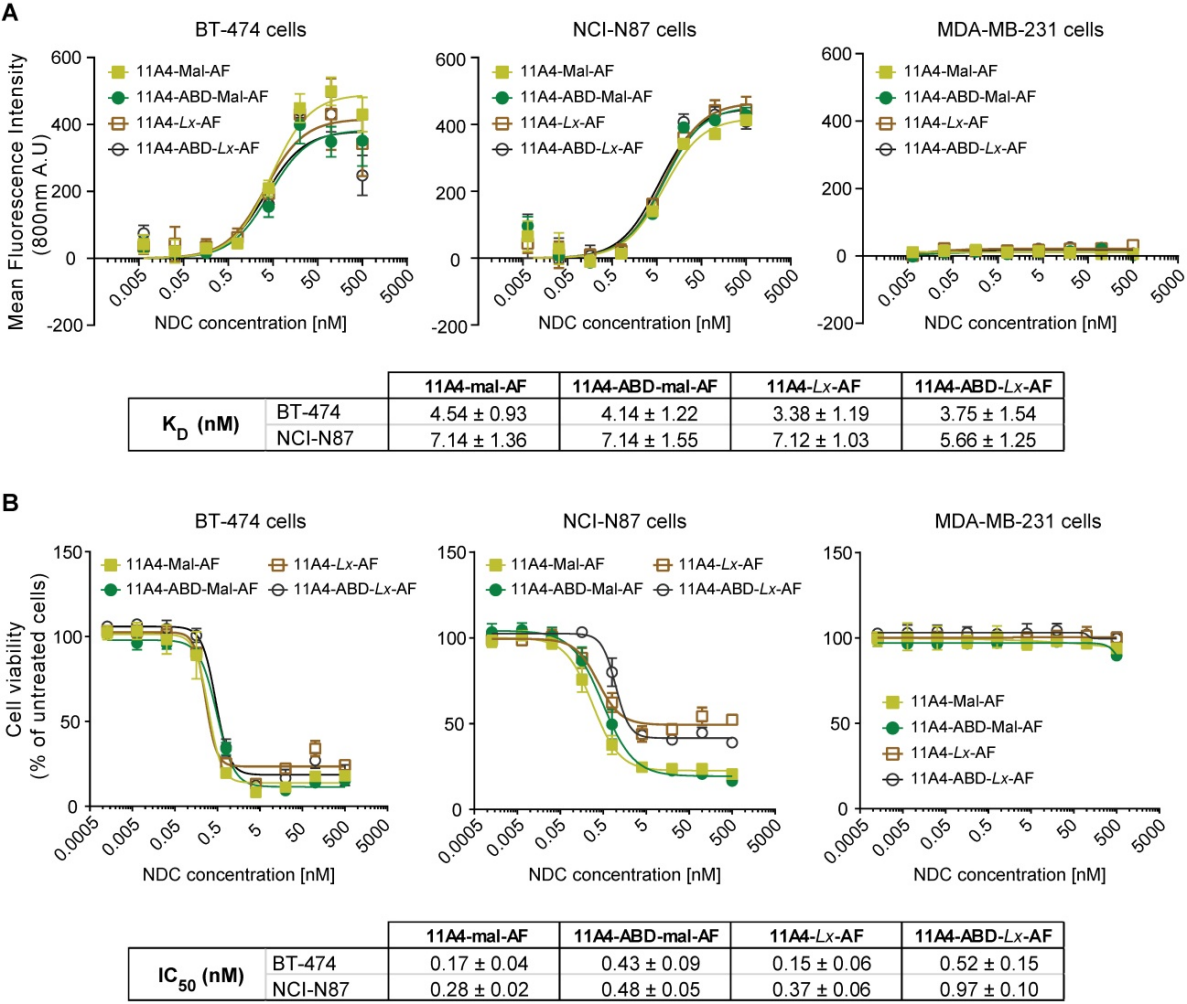
***In vitro* characterization of auristatin F NDCs. A)** Binding assay and **B)** Cytotoxicity assay (5 days incubation) of 11A4-Mal-AF (yellow filled squares), 11A4-ABD-mal-AF (green filled circles), 11A4-Lx-AF (brown open squares) and 11A4-ABD-Lx-AF (black open circles) drug conjugates on HER2-positive cells BT-474 and NCI-N87 cells or HER2-negative MDA-MB-231 cells. Values plotted as mean ± SD (n = 3). The tables display K_D_ and IC_50_ (mean ± SD) as calculated from the graphs in A) and B) respectively.

Auristatin F (AF) was conjugated via the free C-terminal cysteine of the nanobodies using common maleimide-thiol conjugation or the platinum-based *Lx* linker. The obtained AF NDCs presented high purity, a drug-to-antibody ratio (DAR) of 1.0 and the expected molecular weight, as confirmed by SEC-MS analysis (Figure S6). All four 11A4-based NDCs showed specific binding to HER2-positive BT-474 and NCI-N87 cells with nanomolar range K_D_s, but not to HER2-negative MDA-MB-231 (Figure 4A), being comparable to the nanobodies prior to conjugation (Figure S1B). Similar to what was observed for BT-474 cells, both 11A4 and 11A4-ABD were internalized by NCI-N87 cells (k_e,11A4+HSA_: 0.0135 ± 0.0013 min^-1^, k_e,11A4-ABD_: 0.0037 ± 0.0006 min^-1^, k_e,11A4-ABD+HSA_: 0.0036 ± 0.0006 min^-1^, Figure S7), eventually chosen to be used for establishing xenografts for the *in vivo* efficacy testing of the NDCs.

The anti-proliferative effect of both maleimide- and *Lx*-AF conjugated NDCs was evaluated after five days of treatment. Cytotoxicity at low nanomolar concentrations was observed for HER2-positive BT-474 and NCI-N87 cells (Figure 4B) for all HER2-targeted NDCs. At the same time, the viability of HER2-negative MDA-MB-231 cells was not affected, indicating that cytotoxicity is highly target dependent (Figure 4B). Furthermore, treatment of cells with the nanobody alone did not reduce cell viability (Figure S8), supporting that the observed cytotoxicity is attributed to the conjugated AF of the NDC constructs.

### Single dose administration of 11A4-ABD-AF resulted in significant tumor regression

**Figure 5:**
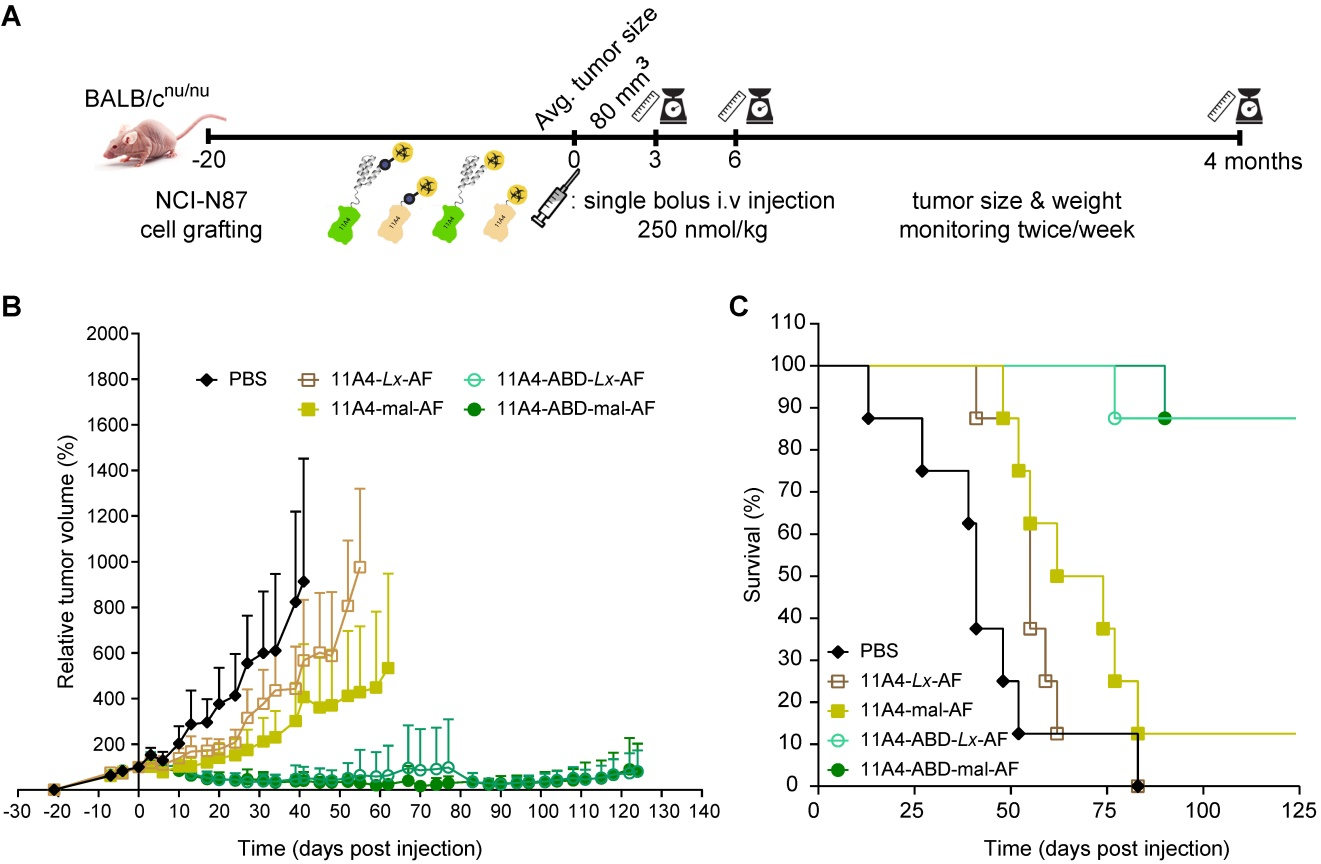
** Therapeutic efficacy of auristatin F NDCs in NCI-N87 tumor bearing mice. Nude mice bearing NCI-N87 tumors in both flanks received a single bolus injection of NDCs with DAR of 1.0 at a dose of 250 nmol/kg. A)** Schematic representation of the therapeutic efficacy study. **B)** Relative tumor volume, expressed as mean ± SD, was measured twice a week for 124 days; and **C)** Kaplan-Meier survival curves of mice injected with 11A4-mal-AF (yellow filled squares, n = 8), 11A4-ABD-mal-AF (green filled circles, n = 8), 11A4-Lx-AF (brown open squares, n = 8) and 11A4-ABD-Lx-AF (green open circles, n = 8) or PBS (black diamonds, n = 8) at day 0. For representation purposes, tumor size of a given group was not included in the graph anymore when animal drop-out was more than 50%. Size of individual tumors can be found in Supplementary Figure S11.

To evaluate the effect that nanobody half-life extension can have on NDC *in vivo* efficacy, mice bearing NCI-N87 xenografts received a single intravenous dose of 250 nmol/kg of 11A4-mal-AF, 11A4-*Lx*-AF, 11A4-ABD-mal-AF or 11A4-ABD-*Lx*-AF. The dose chosen was assumed to be safe, based on previous studies performed with AF-*Lx*-trastuzumab conjugates 42, and was further confirmed after the administration of a single NDC bolus injection at 500 nmol/kg of 11A4-*Lx*-AF (DAR 1.0) and 11A4-ABD-*Lx*-AF (DAR 1.0) in non- tumor-bearing mice (Figure S9). For the efficacy study in tumor bearing mice, control animals received the same volume of PBS as used for the NDC groups (Figure S10A). During the first 10 days, all tumors of NDC treated groups showed growth delay in comparison to the control group (Figure 5A, S11A). In groups that received 11A4-mal-AF or 11A4-*Lx*-AF, tumor regrowth was observed from day 10 onwards. At day 83 of the experiment, all mice of PBS or 11A4-*Lx*-AF were euthanized, as tumor size has reached a maximum, as defined by the humane endpoint of the study (Figure 5C, S11A, S11D). For the group injected with 11A4-mal-AF, only one mouse survived from that day until the end of the study (Figure 5C, S11B). Though, we believe this was not treatment related, as tumors had below average tumor volume and their size had decreased already 3 days p.i. (Figure S11B). Remarkably, in the groups that received the half-life extended NDCs, tumor remission was sustained until day 100. Only a slight tumor size increase, to a volume below the one at the day of NDC administration, was observed during the last 24 days of the study (Figure 5B). Finally, 7 out of 8 mice treated with either 11A4-ABD-mal-AF or 11A4-ABD-*Lx*-AF survived until the end study, at day 124 (Figure 5C, S11C, S11E), without showing any significant weight loss throughout the study, indicating good tolerability of the administered NDCs (Figure S10B).

## Discussion

Antibody drug conjugates are becoming an important category of targeted anti-cancer agents used in the clinic, as supported by the growing number of approved ADCs. However, their non-homogenous intratumoral distribution can often compromise their therapeutic effect. In the present study we showed that fusion of an albumin binding domain to the HER2-targeted nanobody 11A4 extended its serum half-life in mice without compromising its homogenous intratumoral distribution. This half-life extension resulted in the exceptional *in vivo* efficacy of the 11A4-ABD-auristatin F conjugates, already after a single-dose administration. These findings demonstrate that monovalent nanobodies can be effectively used for the development of NDCs for the treatment of solid tumors.

After verifying that fusion of ABD did not affect the binding properties of the nanobody (Figure 1B, S1B), the irrelevant nanobody R2 was exploited to analyze the effect of ABD on nanobody's serum half-life. The high binding affinity to mouse serum albumin (Figure S1C, in agreement with previous data 45), resulted in a 14.8-fold increase in the serum half-life of R2-ABD (44.5 h), compared to R2 (3 h). This corresponds to four times longer half-life compared to the half-life reported for an irrelevant nanobody fused to an anti-albumin nanobody (τ^β^_1/2, Irr1-Hle1_: 13.8 h), although that one had a considerably lower affinity for albumin 52. Organ-radioactivity levels of ^125^I-R2-ABD were lower than those in the blood. This implies absence of non-specific ^125^I-R2-ABD organ retention, suggesting that albumin-binding per se is the dominant factor mediating its slower clearance. This is further supported by the fact that the observed half-life of R2-ABD is similar to the measured half-life of radioiodinated albumin in mice (⁓39h) 53. Assuming that nanobody-ABD clearance will follow that of human serum albumin, we anticipate that its half-life in humans will be close to 19 days and thus closer to that of human IgGs 54.

In addition, tumor accumulation of 11A4-ABD was also prolonged, as was previously observed for other antibody fragments and nanobodies fused to albumin binding moieties 55-57. For 11A4, the highest tumor fluorescence intensity was observed already 1 h post injection (p.i.), being in agreement with previously reported data 44. Indicative of the difference in pharmacokinetics, tumor fluorescence signal of 11A4-ABD showed a more gradual increase, reaching and maintaining a maximum from 5 to 24 h p.i. *In vivo* molecular imaging and biodistribution revealed reduced kidney retention for both targeted and irrelevant ABD-fused nanobodies. Concomitantly, the liver content increased for the nanobody-ABD constructs indicating that renal clearance partly shifted to catabolism at the liver. Such a drastic decrease in renal clearance and retention was not documented for neither the bivalent construct (aTNF-aTNF-aAlb, serving as non-targeting control of that study 56) nor for anti-HGF monovalent nanobodies 58, both employing anti-albumin nanobodies (anti-Alb) instead of an ABD peptide for half-life extension. However, in those studies the hexa-histidine purification tag (6x His) was not removed prior to *in vivo* administration. In general, kidney retention can depend on the charge of the molecule, and has been suggested to be megalin mediated 59, with IRDye800CW-labelled nanobodies reported to have extended kidney retention 60. The EPEA tag used in the present study could be offering an advantage over the highly charged 6× His tag of the anti-Alb-containing constructs, that has been shown to result in increased kidney retention 61. Nevertheless, it cannot be excluded that the albumin dissociation constants of ABD and anti-Alb nanobody could also influence this. A direct comparison of such constructs is necessary to clarify the contribution of the purification tag and of the albumin binding moiety in kidney retention. Overall, based on our observations, the use of EPEA tagged ABD-fused constructs can be considered advantageous when designing NDCs, as it could decrease the accumulation of an NDC in the kidneys and thus minimize the risks of nephrotoxicity.

Furthermore, our *in vivo* imaging study shows that the transient increase in the hydrodynamic radius of 11A4-ABD did not affect its homogeneous intra-tumoral distribution *in vivo*. This was already anticipated, as 11A4 and 11A4-ABD had comparable diffusion patterns in HER2-expressing 3D spheroids that have been proposed to be a good *in vitro* model to evaluate *in vivo* distribution 49. Consequently, 11A4-ABD could be expected to maintain the intra-tumoral distribution advantage demonstrated in *in vivo* imaging studies of monovalent HER2-targeted nanobodies over trastuzumab 50.

As expected from previous studies with AF-trastuzumab 42, our NDCs demonstrated high cytotoxicity only against HER2-expressing cells. NDCs were able to bind and internalize, with internalization rate constants (k_e_) being in the same range of what has been described for HER2 and HER2-targeted nanobodies 34,46,62, therefore allowing for cytoplasmic drug delivery. No significant differences were seen between the IC_50_ of 11A4-AF and 11A4-ABD-AF*,* after 5 days of treatment* in vitro*. This observation was in agreement with the similar k_e_s of 11A4 and 11A4-ABD, that could eventually be translated in comparable cytosolic concentrations of the delivered AF after 5 days.

We finally tested the *in vivo* efficacy of AF NDCs in mice bearing xenografts established after inoculation of HER2-positive NCI-N87 cells. A single bolus injection of 250 nmol/kg of 11A4-ABD-AF demonstrated excellent efficacy *in vivo*. Durable remissions (>124 days) were observed with 11A4-ABD-AF conjugates - much longer than with 11A4-AF conjugates - irrespective of whether maleimide or *Lx* conjugation was used. In total 7 out of 8 mice treated with either 11A4-ABD-mal-AF or 11A4-ABD-*Lx*-AF survived until the end of the study at day 124. These therapeutic results are very similar to results previously obtained in the same xenograft model with trastuzumab-mal-AF and trastuzumab-*Lx*-AF 42.

The similar efficacy of the 11A4-ABD-AF and trastuzumab-AF conjugates is remarkable when taking into account the tumor uptake levels. In the present therapy studies, 11A4-ABD-AF was used at an AF dose of 250 nmol/kg. Based on the imaging studies, the tumor uptake levels of 11A4-ABD could possibly be in the range of ~6 %ID/g at 3 days after injection. After administration of a single bolus injection of trastuzumab-AF at a dose of 15 mg/kg and a DAR of 2.6, translating to roughly 260 nmol/kg, Sijbrandi and colleagues have reported trastuzumab-AF tumor uptake levels of 31.0 ± 6.0 %ID/g, 4 days p.i. 42. These combined results suggest that the lower tumor uptake of 11A4-ABD-AF, as opposed to trastuzumab-AF, seems to be compensated by the more homogeneous tumor distribution of the NDC, resulting in similar efficacy for both types of constructs. Further MTD and efficacy studies with 11A4-ABD-AF, and side-by-side comparison with trastuzumab-AF, are needed to assess which of the two constructs has the most favorable therapeutic potential.

Interestingly, trastuzumab has been shown to localize in brain metastasis both in pre-clinical models 35 and in patients 63, something most probably explained by the presence of a fenestrated endothelium in brain lesions. Puttemans et al reported higher tumor accumulation for the HER2-targeted nanobody [^111^In]-2Rs15d (1h p.i.) compared to [^111^I]-Trastuzumab (3d p.i.) administered in a mouse model bearing intracranial SK-OV-3 tumors, suggested to have less disrupted BBB. Hence, it would be of great interest to compare small-sized ABD-fused NDCs with trastuzumab or trastuzumab-based ADCs also in the context of HER2-expressing brain metastases treatment, which are frequently linked with poor prognosis for breast cancer patients.

The model used in the present study is based on a highly expressed tumor target. Yet, targets with lower expression or with very slow internalization rates could be employed instead. In these cases, the use of bivalent NDCs should be considered in order to stimulate more efficient cytosolic delivery of the conjugated drug. However, increased internalization rate, along with high binding affinity and valency (linked with a low k_off_) can slow down or even completely prevent diffusion towards the center of the tumor mass 64,65. The choice of an ABD over anti-Alb for half-life extension, can prevent a further increase in the size of dimeric constructs. Such a size increase could potentially affect their tumoral distribution, as suggested by studies where fusion to a non-targeting nanobody has been employed to account for the size effect 49,50. Along these lines, in one of the very few examples of a solid tumor targeting NDCs, a cisplatin conjugated biparatopic anti-EGFR nanobody construct fused to anti-Alb was not able to demonstrate comparable tumor remission 66. The tumor model used as well as differences in the dosing, payload (repeated administration of an a NDC with 3× higher DAR versus single administration of a dose twice as high used here) and physicochemical characteristics compared to the conjugates used in the current study could be greatly affecting their efficacy. A direct comparison between the distribution of these two different NDC formats would be very interesting to explore experimentally, in order to also evaluate how different half-life extension strategies (i.e. anti-Alb nanobody versus ABD) can be affecting NDC efficacy.

Recently, Nessler et al (2020) reported equally promising results using single-domain antibody-based drug conjugates in a prostate cancer model 21. As demonstrated in penetration studies performed in spheroids, as well as histological analysis of tumors, monovalent prostate-specific membrane antigen (PSMA)-targeting Humabodies^®^ (fully human heavy-chain variable domain antibodies derived from the Crescendo mouse) were able to distribute more homogeneously compared to bivalent Humabodies or the monoclonal antibody used as reference in the study. Anti-PSMA Humabody conjugates were not able to demonstrate comparable remissions in a tumor model of high expressing cells (⁓10^6^ receptors/cell), which is similar to the HER2 expression levels of the NCI-N87 cells in this study. On the other hand, the monomeric serum half-life-extended Humabody-drug conjugate demonstrated excellent efficacy in the moderate PSMA expression (<10^5^ receptors/cell) xenograft model, already after single dose administration of a considerably lower dose compared to that of 11A4-ABD-AF. Even though a direct comparison cannot be performed, as different target and payload are used, both studies are highlighting that homogenous targeting of a larger population of cells, by means of smaller monovalent NDCs, can correlate with better efficacy when sufficient exposure is guaranteed.

In conclusion, we have shown that nanobodies can be successfully used as a platform for the development of drug conjugates that can offer more complete tumor targeting. Of key importance for their *in vivo* efficacy is the extension of their serum half-life. Herein, we demonstrated that half-life extension, by the fusion of an ABD to the C-terminus of an anti-HER2 nanobody, resulted in prolonged and homogeneous tumor accumulation, accompanied by reduced kidney retention. A single dose administration of internalizing monovalent AF conjugates targeting HER2 resulted in excellent efficacy in a model of HER2 overexpressing tumors *in vivo*. This demonstrates the great potential of such an approach, encouraging further testing in different tumor models.

## Methods

An in-depth description of production, purification and functionalization of nanobodies, as well as of the assays used for their *in vitro* characterization can be found in the Supplementary Materials and Methods.

### Cell lines

All cancer cell lines used in this study were obtained from the American Type Culture Collection (ATCC) (LGC Standards, Germany). The HER2-positive invasive ductal carcinoma cell line BT-474 (ATCC^®^ HTB-20^™^) and the HER2-negative adenocarcinoma MDA-MB-231 (ATCC^®^ HTB-26^™^) were maintained in high glucose Dulbecco's Modified Eagle's Medium (DMEM 4.5 g/l glucose L-Glutamine; Lonza Benelux BV, Breda, the Netherlands), while the HER2-positive gastric carcinoma NCI-N87 cells (ATCC^®^ CRL-5822^™^) were cultured in RPMI-1640 medium (L-Glutamine, 25 mM HEPES, Gibco^™^; Thermo Fisher Scientific Inc, Breda, the Netherlands). All media were supplemented with 10% (v/v) fetal bovine serum (FBS; GE Healthcare Europe GmbH, Eindhoven the Netherlands), 100 U/ml penicillin and 100 μg/ml streptomycin (Sigma-Aldrich BV, Zwijndrecht, the Netherlands). Cells were kept at 37 °C in a humidified atmosphere containing 5% CO_2_ and were constantly tested negative for Mycoplasma.

### Nanobody constructs

Codon optimized genes encoding for the HER2-targeted nanobody 11A4 44 and the irrelevant nanobody R2 67, fused to the streptococcal protein G ABD sequence 39 were purchased from Integrated DNA Technologies (IDT BVA, Leuven, Belgium) and ligated into a pET28a vector provided with a C-terminal cysteine and EPEA affinity purification tag.

### Nanobody production, purification, iodination and fluorophore conjugation

Nanobodies were produced and affinity purified from the periplasmic space of BL21-CodonPlus (DE3)-RIL *E. coli* bacteria employing the EPEA tag. Sample's purity was analyzed with 15% SDS-PAGE and stored at -20 °C until further use.

For radiolabeling of nanobodies the Iodogen method was used 68. Nanobodies were labeled at their C-terminal cysteine with the near infrared fluorophore IRDye800CW (IR)-maleimide or Alexa488-maleimide. Human Serum Albumin (HSA) was randomly conjugated to NHS-Alexa647. The amount of free fluorophore was below 5% in all samples.

### Auristatin F nanobody conjugate synthesis

Free C-terminal cysteine-containing 11A4 and 11A4-ABD were used for conjugation to auristatin F either via maleimide or employing the *Lx* linker technology following the procedure described in detail in the Supplementary methods. All samples were subjected to SDS-PAGE and SEC-MS analysis to evaluate their purity and integrity prior to use *in vivo*.

### Binding affinity determination on cells

Binding affinity determination of nanobodies and NDCs on BT-474, NCI-N87 and MDA-MB-231 cells was done as described previously 44. Essentially, a dilution series of the different constructs were incubated with cells for 2 h at 4^o^C, in the presence or absence of HSA. Bound nanobodies were either directly detected via measuring the IR fluorescence intensity of nanobody conjugates or indirectly detecting NDCs using an anti-VHH antibody.

### Evaluation of interaction with serum albumin

An ELISA on immobilized serum albumin from different species was used to determine the binding affinity of ABD-containing nanobodies for albumin. To evaluate whether albumin competes for binding on HER2 expressing cells, a constant concentration of directly conjugated 11A4-ABD was incubated with cells in the presence of varying amounts of BALB/c mouse plasma or HSA.

### Evaluation of nanobody internalization in cells

Internalization of the different nanobody constructs was measured after incubation of a constant concentration of IR-conjugated samples with HER2 expressing cells at 37 °C for a period of 15 min. Total fluorescence as well as fluorescence attributed to the internalized fraction were measured and used to calculate the internalization rate constant as described by Heukers et al. 69.

### *In vitro* cell viability assay

The effect of auristatin F nanobody conjugates on the *in vitro* cell viability was measured using the AlamarBlue^®^ Reagent, following provider's protocol.

### Spheroid 3D cell culture and immunofluorescence staining

Three dimensional tumor cell spheroids of BT-474 cells were cultured on top of a Matrigel^®^ scaffold and allowed to grow up to a size with a radius below 200 μm. Spheroids were treated for different time intervals with Alexa488 fluorescently labeled nanobodies in the presence of HSA-Alexa647. Paraformaldehyde-fixed samples were imaged using confocal laser scanning microscopy.

### Determination of percentage of radius of area covered by the fluorescent proteins

Spheroid images were acquired at a z-plane at the middle of the spheroid. Using ImageJ, obtained images were thresholded just above background. The thresholded area, corresponding to the area covered by the diffusing probe, as well as the total spheroid area were used to calculate the probe's displacement. See Supplementary Material and Methods for a detailed description.

### Evaluation of *in vivo* half-life

All animal experiments were carried out in accordance with the National Institutes of Health Principles of Laboratory Animal Care and Dutch national law (“Wet op de dierproeven”, Staatsblad 1985, 336).

For the determination of the nanobody half-life in blood circulation, two different groups of four female nude mice (BALB/cOlaHsd-*Foxn1^nu^*) received intravenously 2.8x10^7^ cpm of either R2 (12.5 μg) or R2-ABD (17.5 μg) ^125^I-labeled nanobody. Blood samples (10 μl) were withdrawn through the vena saphena at 3 min, 30 min, 1, 3, 7, 24, 48, 72 and 96 h post injection (p.i.) and radioactivity levels were immediately measured with a Packard Cobra II γ-counter (PerkinElmer). The percentage of injected dose (%ID) was calculated and plotted as μg per mL of blood over time. A curve was fitted with a two-phase decay model and terminal half-life [τ^β^_1/2_ (hours)] as well as Area Under the Curve (AUC), Clearance [CL (mL/time) = Dose (μg)/AUC (μg/mL•min)], Rate of elimination [k_e_ (h^-1^) = ln2/τ^β^_1/2_] and Volume of distribution = [V_d_ (mL) = CL/k_e_] were calculated.

### *In vivo* optical molecular imaging experiments

For the optical imaging study female nude mice (BALB/cOlaHsd-*Foxn1^nu^*) bearing BT-474 xenografts of an average size of 79 mm^3^ received intravenously 50 μg of a) 11A4-ABD-IR (n = 4), b) R2-ABD-IR (n = 4) and c) 11A4-IR (n = 2). Mice with MDA-MB-231 tumors received 50 μg of a) 11A4-ABD-IR (n = 4) or b) R2-ABD-IR (n = 4). For imaging, mice were anesthetized and images were acquired before administration and 1, 2, 5, 24, 48 and 72 h p.i. using Pearl^®^ Impulse Small Animal Imaging System (LI-COR^®^ Biosciences, Westburg BV, Leusden, the Netherlands). Images were analyzed using Pearl Impulse Software v2.0 (LI-COR^®^ Biosciences) were regions of interest (ROI; same size within each mouse) were drawn around the tumors and at normal tissue areas (background), fluorescent intensity per pixel was calculated and plotted over time. After the last imaging time point, mice were sacrificed and organs as well as tumors were collected. Tissue biodistribution of IR-probes as well as histological analysis of formalin fixed tumors were performed as described in supplementary Materials and Methods.

### *In vivo* safety and efficacy experiments

In order to evaluate possible systemic toxicity of the auristatin-F NDCs, two groups of 5 animals each (female 6-8 weeks old athymic Nude-*Foxn1^Nu^* mice) received retro-orbitally a single 8 mg/kg dose of either 11A4-*Lx*-AF or 11A4-ABD-*Lx*-AF. Weight was monitored daily for a total of 14 days. Safe dosing was determined as one where body weight reduction at two consecutive time points was not more than 10% relative to the body weight at the start of the experiment.

For the efficacy study, mice bearing subcutaneous NCI-N87 tumors on both flanks (average tumor size of 80 mm^3^) were randomly grouped (8 mice per group) and received a single bolus dose of NDCs at 250 nmol/kg (dose volume: 100 μL per 25 g) by retro-orbital injection. Groups were treated with either a) 11A4-Mal-AF or b) 11A4-*Lx*-AF or c) 11A4-ABD-*Lx*-AF or d) 11A4-ABD-Mal-AF or PBS, which served as the vehicle control. Tumor volume as well as body weight were measured twice a week over a period of 124 days after injection. Individual animals were monitored until a humane endpoint was reached (among others tumor size greater than 1000 mm^3^ and acute irreversible weight loss). Tumors between 50 and 300 mm^3^ at the start of treatment were included in the final tumor regression calculations.

### Statistical analysis

The statistical analysis of the tissue biodistribution was performed using Welch's t-test of unpaired samples. A two-tailed significance was calculated, and P values < 0.05 were considered statistically significant.

## Supplementary Material

Supplementary materials and figures.

## Abbreviations

%ID/g: percentage of injected dose per gram of tissue
A488: AlexaFluor488
A647: AlexaFluor647
ABD: albumin binding domain
ADC: antibody drug conjugate
AF: auristatin F
anti-Alb: anti-albumin nanobody
AUC: area under curve
BSA: bovine serum albumin
CL: clearance
DAR: drug to antibody ratio
DoC: degree of conjugation
h: hour
H&E: hematoxylin and eosin
HSA: human serum albumin
IR: IRDye800CW
K_D_: dissociation constant
mAb: monoclonal antibody
MSA: mouse serum albumin
MTD: maximum tolerated dose
NDC: nanobody drug conjugates
p.i.: post injection
RT: room temperature
SEC: Size-Exclusion Chromatography
V_d_: volume of distribution
τ^β^_1/2_: terminal half-life
