# Supplementary material for: Homogeneous tumor targeting with a single dose of HER2-targeted albumin-binding domain-fused nanobody-drug conjugates results in long-lasting tumor remission in mice
Source: Theranostics. 2021 Mar 13;11(11):5525–38. doi: 10.7150/thno.57510 (PMC8039960; doi:10.7150/thno.57510)
Supplement: Supplementary file 1 — Supplementary materials and figures. [file thnov11p5525s1.pdf]

Homogeneous tumor targeting with a single dose of HER2-targeted nanobody-drug conjugates results in long-lasting tumor remission in mice

Katerina T Xenaki[1], Bram Dorresteijn[1], Joey A Muns[2], Kevin Adamzek[2], Sofia Doulkeridou[1], HendrikJan Houthoff[2], Sabrina Oliveira[1,3#], Paul MP van Bergen en Henegouwen[1]

[1] Cell Biology, Neurobiology and Biophysics, Department of Biology, Faculty of Science, Utrecht University, Utrecht, The Netherlands
[2] LinXis B.V., Amsterdam, The Netherlands
[3] Pharmaceutics, Department of Pharmaceutical Sciences, Faculty of Science, Utrecht University, Utrecht, The Netherlands
[#]correspondence: s.oliveira@uu.nl; Tel.: +31634103460

Supplementary figures

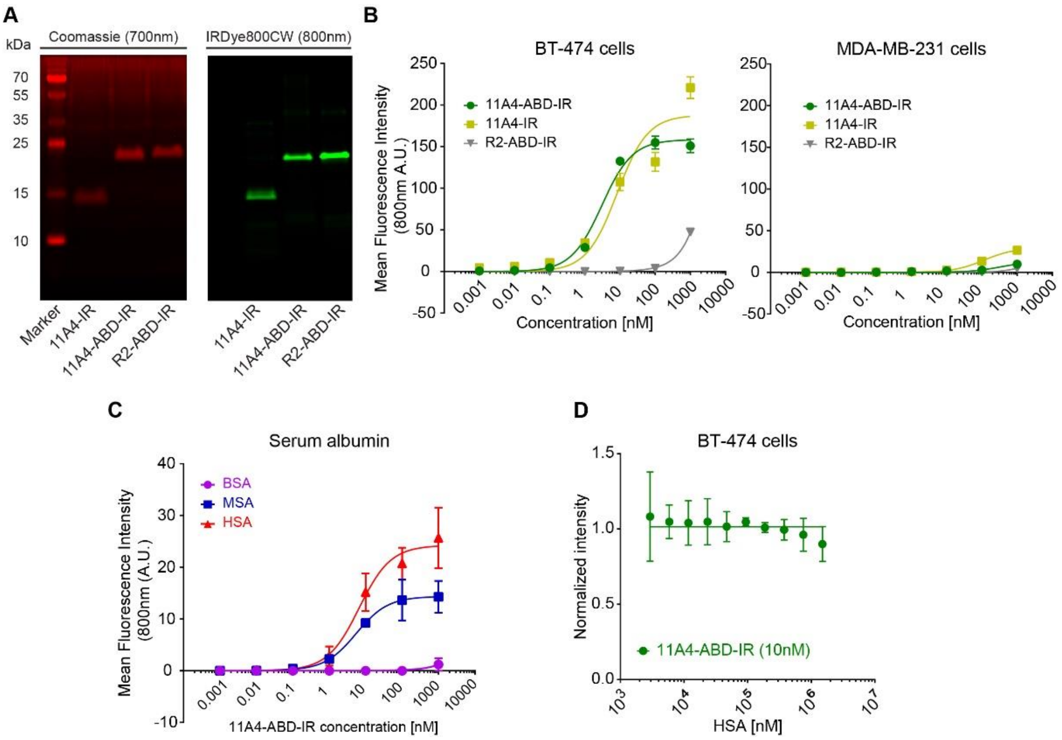

Figure S1 – Characterization of ABD-fused nanobodies. A) Representative SDS-PAGE analysis of IRDye800CW (IR) conjugated nanobodies. Gel was stained with Coomassie blue stain and scanned at 700 nm (Coomassie) and 800 nm (IR). **B)** Binding assay of 11A4-IR (yellow squares), 11A4-ABD-IR (green circles) and control R2-ABD-IR (grey triangle) on BT-474 (HER2-positive) or MDA-MB-231 (HER2-negative) cells. **C)** Binding ELISA of 11A4-ABD-IR on immobilized serum albumin of different origin. Bovine Serum Albumin (BSA; magenta circles), Mouse Serum Albumin (MSA ; blue squares) and Human Serum Albumin (HSA; red triangles). **D)** Competition binding of 11A4-ABD-IR on BT-474 cells in the presence of HSA. Values were plotted as mean ±SD (n = 3).

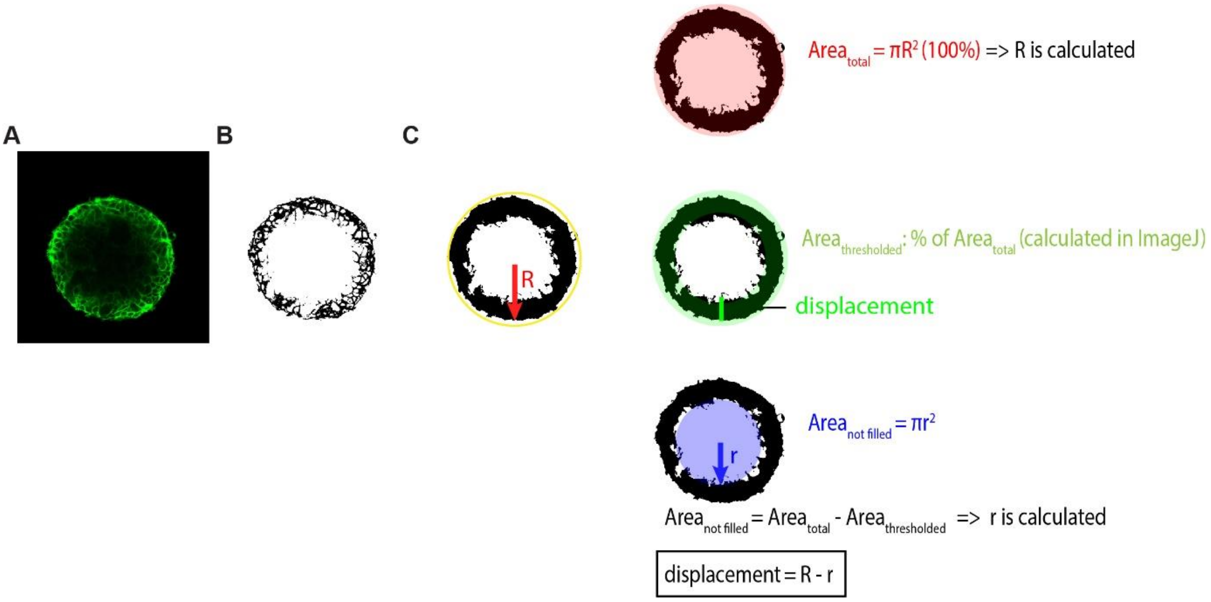

Figure S2 – Calculation of fluorescent probe displacement in *in vitro* 3D spheroids. A) BT-474 spheroids incubated with Alexa488-conjugated nanobodies were fixed at different time points. Samples were imaged by confocal microscopy at a z-plane around the middle of each spheroid. **B)** Images were thresholded just above background using ImageJ, obtaining a binary image with the area that probes had covered. **C)** Thresholded area was then dilated, to fill the intracellular space of cell layers where bound probe was detected. The perimeter of the spheroid was selected and used to determine the spheroid's total surface (red; $Area_{total}$) and the radius (R). The surface of the thresholded (green; $Area_{thresholded}$) was determined using ImageJ. The area still not penetrated by the fluorescent nanobodies (blue; $Area_{not\text{-}filled}$) was calculated. From this information, the radius of $Area_{not\text{-}filled}$ (r) was calculated and used to determine the displacement of the fluorescent probe ((R-r)/R. 100%).

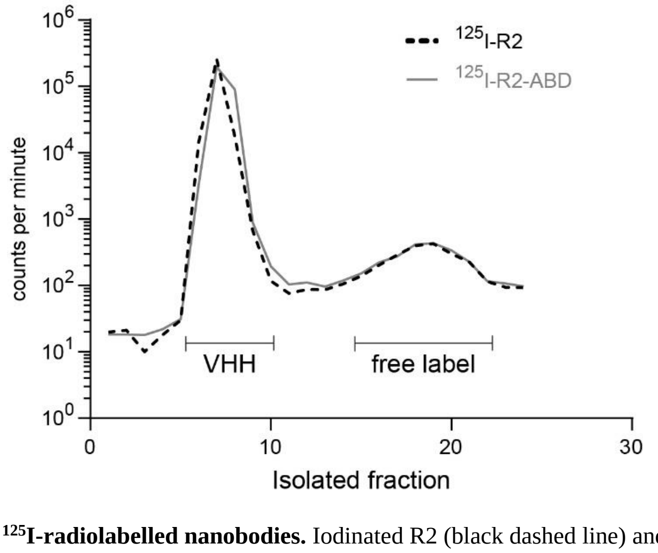

Figure S3 – Purification of ^{125}I-radiolabelled nanobodies. Iodinated R2 (black dashed line) and R2-ABD (grey continuous line) were separated from the unreacted radiolabel by means of Size Exclusion Chromatography prior to their *in vivo* use. The radioactivity of individual fractions was further determined, to identify nanobody-containing fractions.

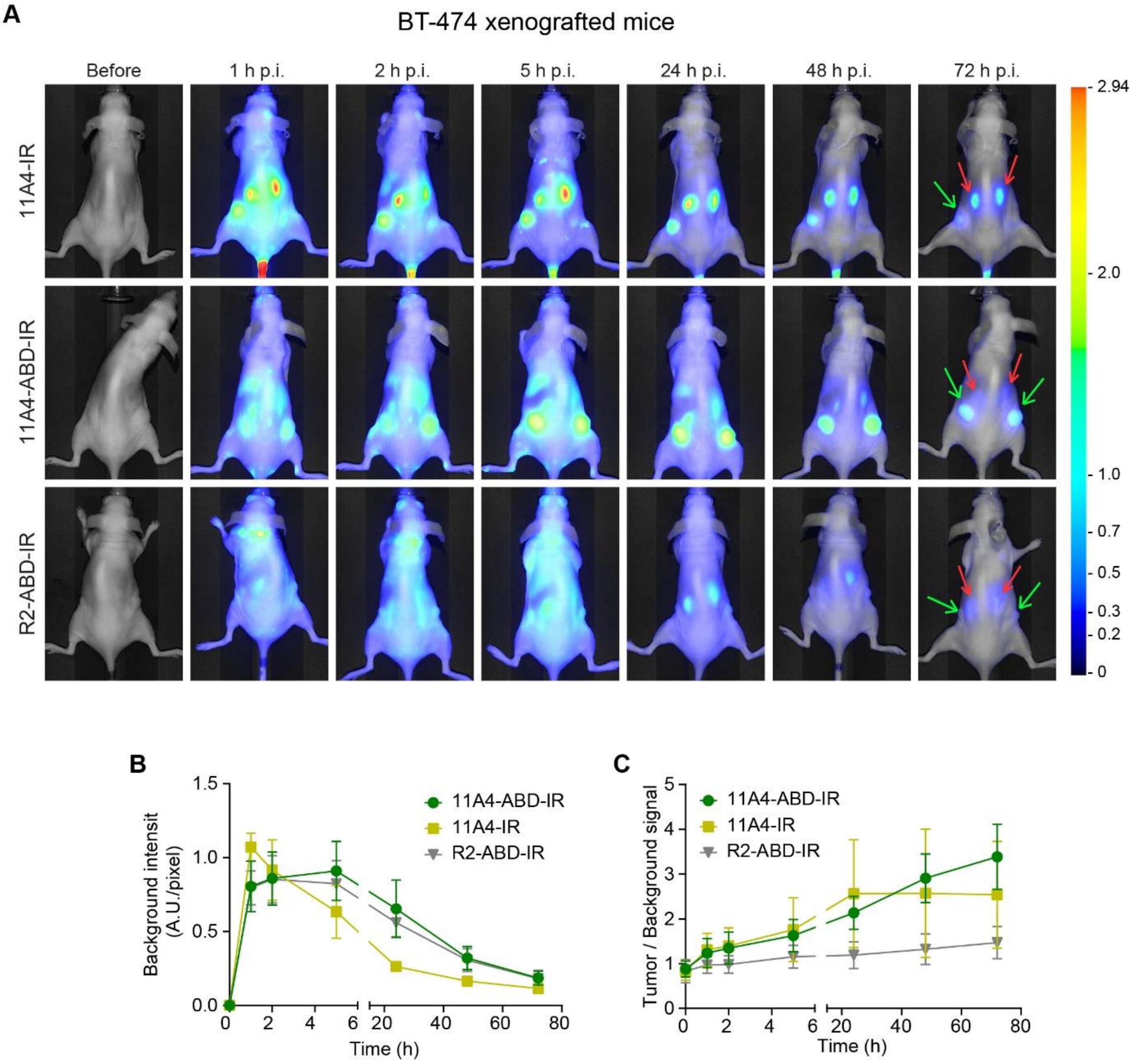

Figure S4 – *In vivo* imaging of BT-474 xenografted nude mice. A) Representative images of BT-474 tumor-bearing mice after single dose administration (50 µg/ mouse) of IR labelled probes. Red arrow: kidney and green arrow: tumors. **B)** Fluorescence intensity measured at a non-tumor area away from the kidneys (skin region of the pelvic area; background) was plotted over time. **C)** Tumor-to-background ratio of fluorescent intensity was determined for all probes. Values were plotted as mean ± SD.

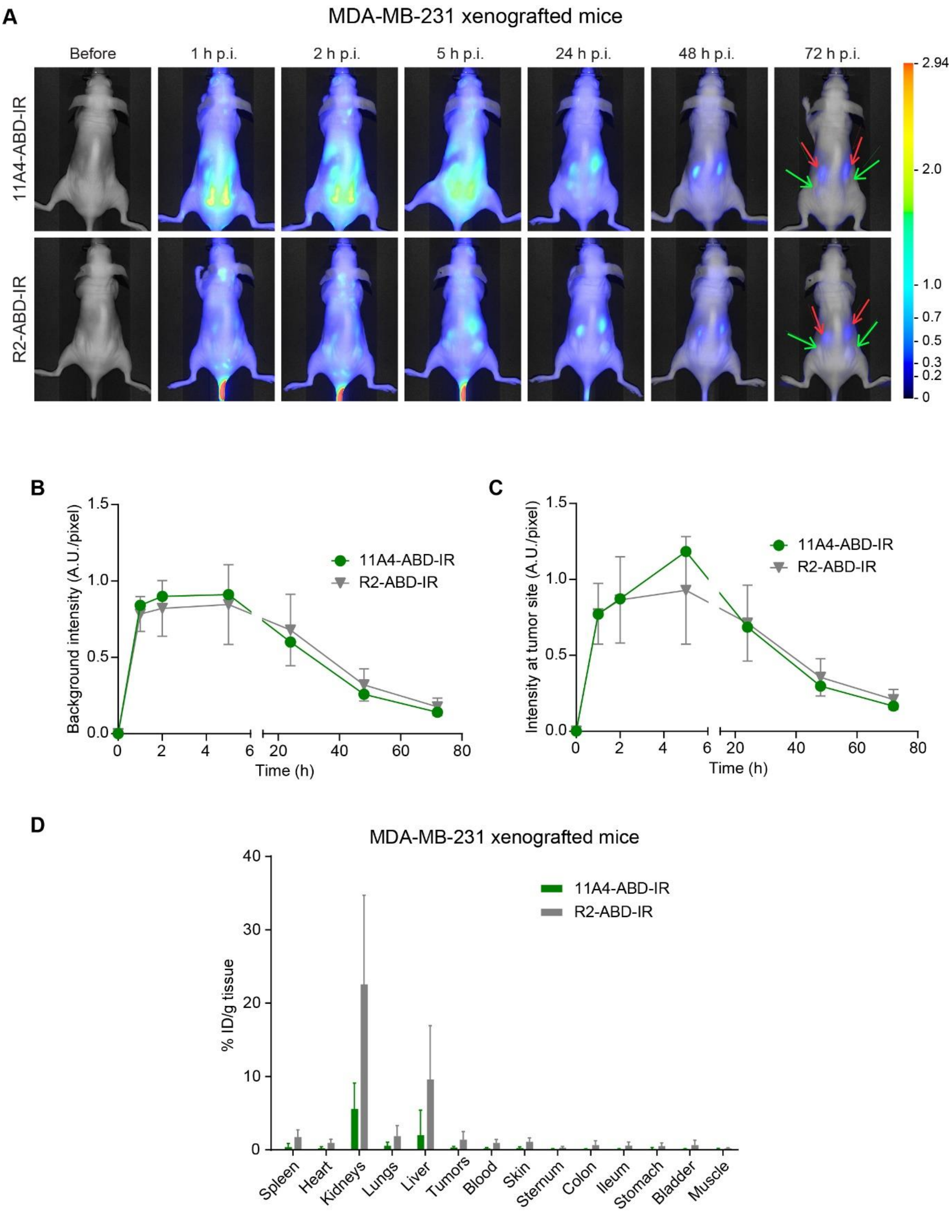

Figure S5 - *In vivo* imaging of MDA-MB-231 xenografted nude mice. A) Representative images of MDA-MB-231 tumor-bearing mice after single dose administration (50 µg/ mouse) of IR labelled probes. Red arrow: kidney and green arrow: tumors. Fluorescence intensity measured at the tumor **(B)** or at a non-tumor area **(C)** were plotted over time. **D)** Tissue biodistribution was performed 4 days after the administration of the IR probes. Values were plotted as mean ± SD.

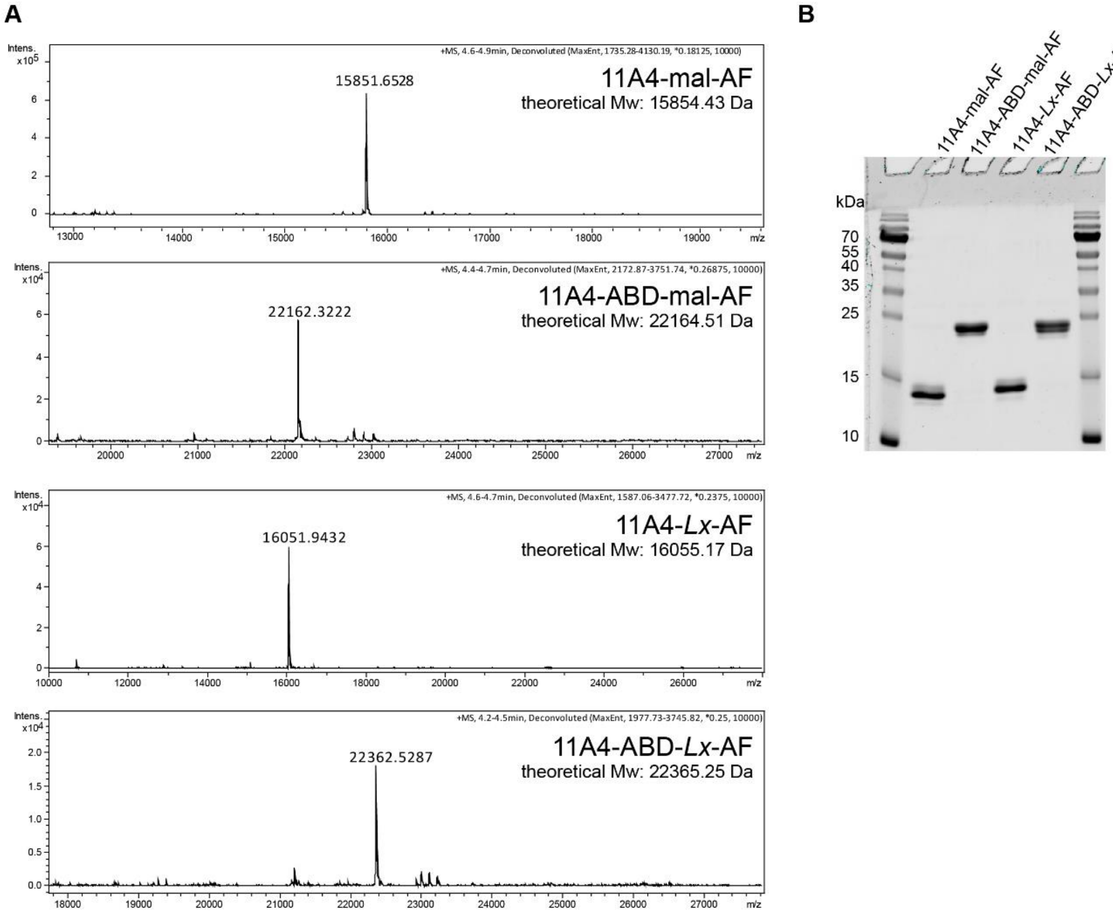

Figure S6 – Determination of Drug-to-Antibody ratios and NDC purity. A) Deconvoluted mass spectra obtained upon SEC-MS analysis of the different auristatin F conjugates and the calculated molecular weights. **B)** SDS-PAGE analysis of NDCs just before *in vivo* administration.

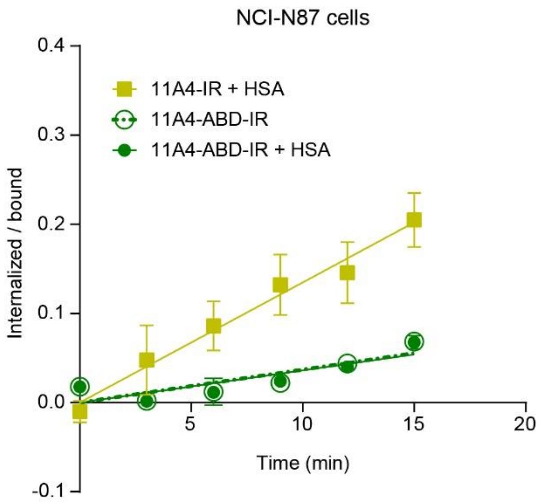

Figure S7 – Internalization by HER2-positive NCI-N87 cells. Ratio of internalized over bound signal of 11A4-IR (yellow squares) or 11A4-ABD-IR in the absence (open green circles, dashed lines) or presense of HSA (filled green circles, continuous lines) over time on NCI-N87 cells. Values were plotted as mean ± SD (n = 3).

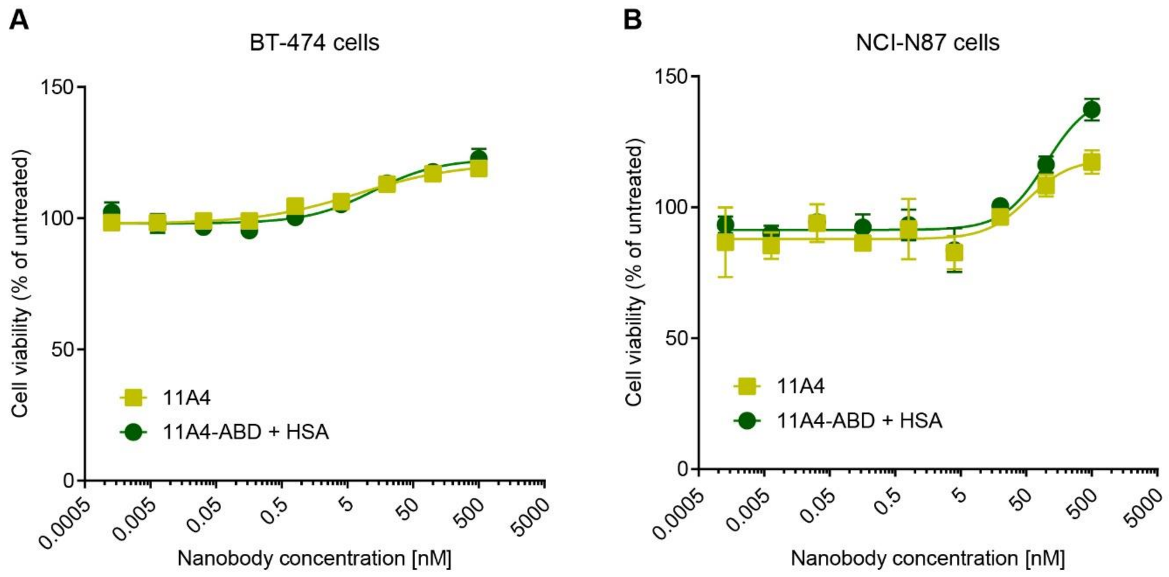

Figure S8 – Cytotoxicity assay on HER2-positive cells. Viability of **A)** BT-474 and **B)** NCI-N87 cells treated with unconjugated nanobodies 11A4 (yellow filled squares) or 11A4-ABD (green filled circles) in the presence of HSA. Values plotted as mean ± SD (n = 3).

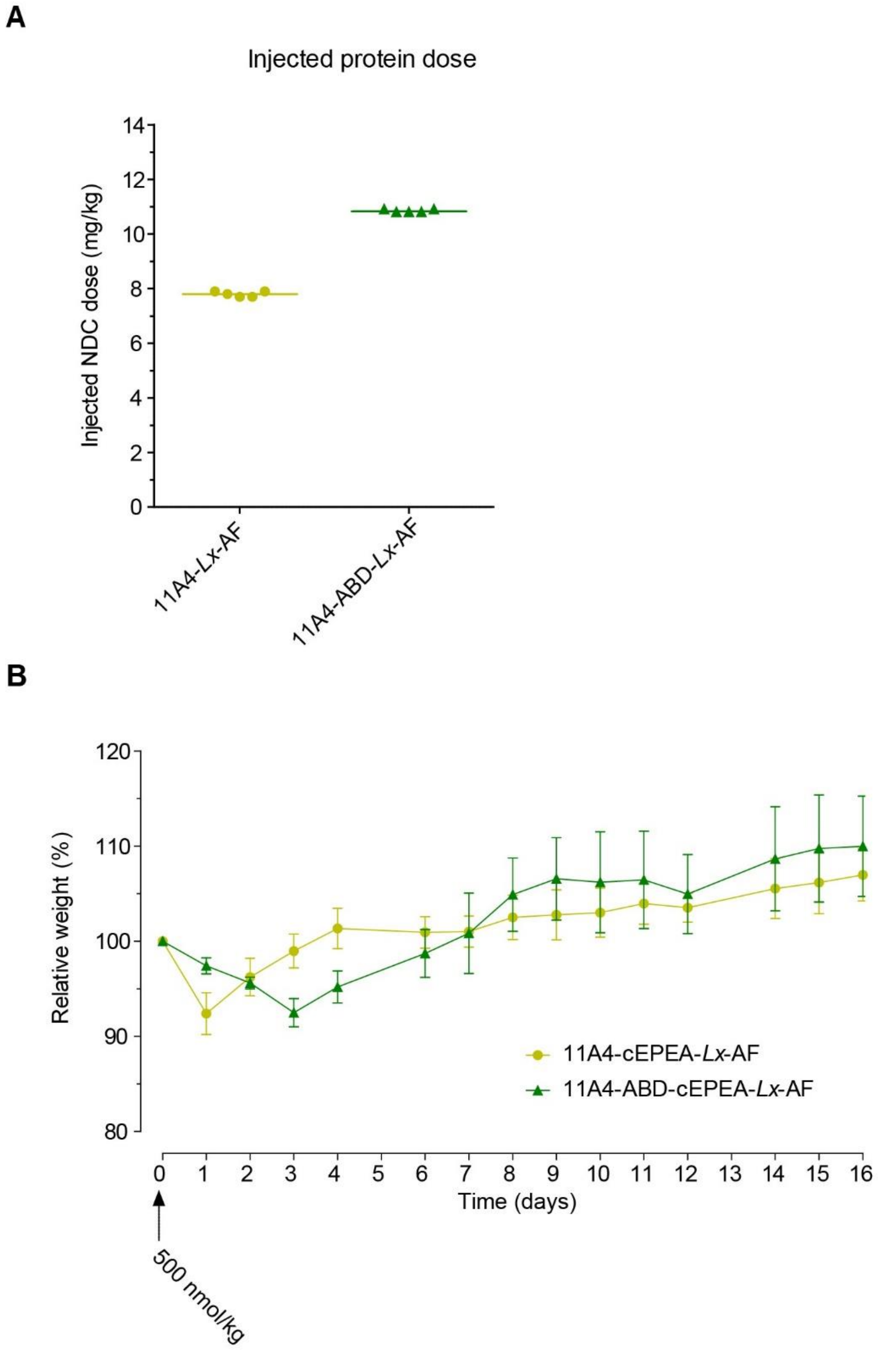

Figure S9 – *In vivo* safety of auristatin F NDCs. Nude mice received intravenously a single dose of auristatin F conjugated via the *Lx* linker to nanobodies. **A)** Administered dose of 500 nmol/kg of either 11A4 (approx. 8 mg/kg NDC dose) or 11A4-ABD (approx. 11.2 mg/kg NDC dose) conjugates. **B)** Animal weight was monitored daily for 16 days post injection. Values were plotted as mean ± SD (n = 5).

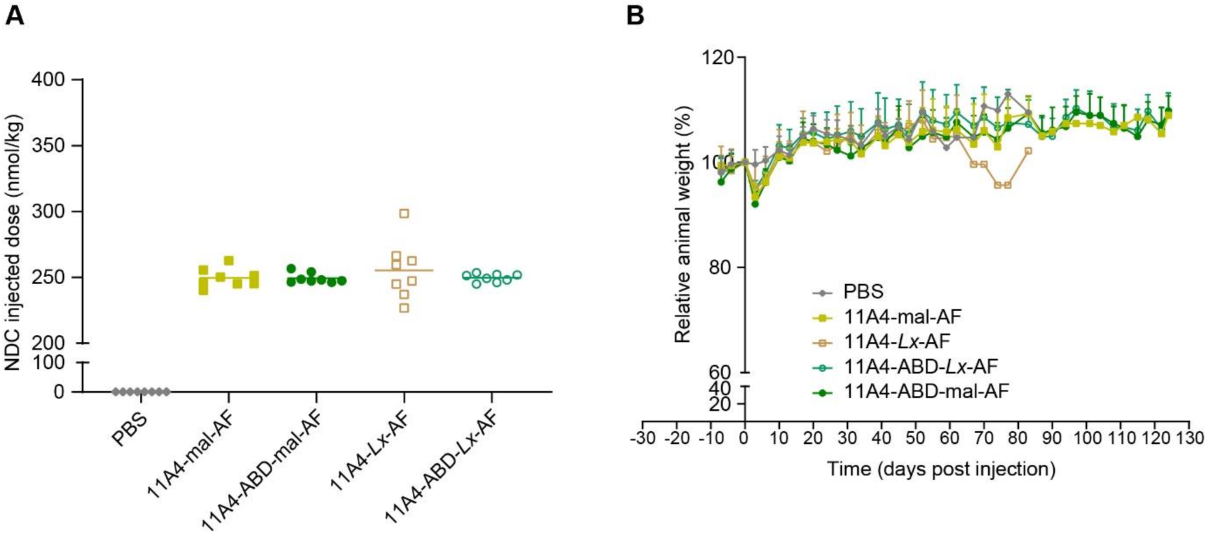

Figure S10 - *In vivo* efficacy study of auristatin F NDCs. NCI-N87 tumor-bearing mice received intravenously a single 250 nmol/kg dose of auristatin F conjugated nanobodies. **A)** Administered dose of either maleimide or *Lx* 11A4 and 11A4-ABD conjugates. **B)** Animal weight was monitored twice a week during the study (124 days). Values were plotted as mean ± SD (n = 8 mice). For representation purposes, the weight of a given group was not plotted anymore when animal drop-out was more than 50%.

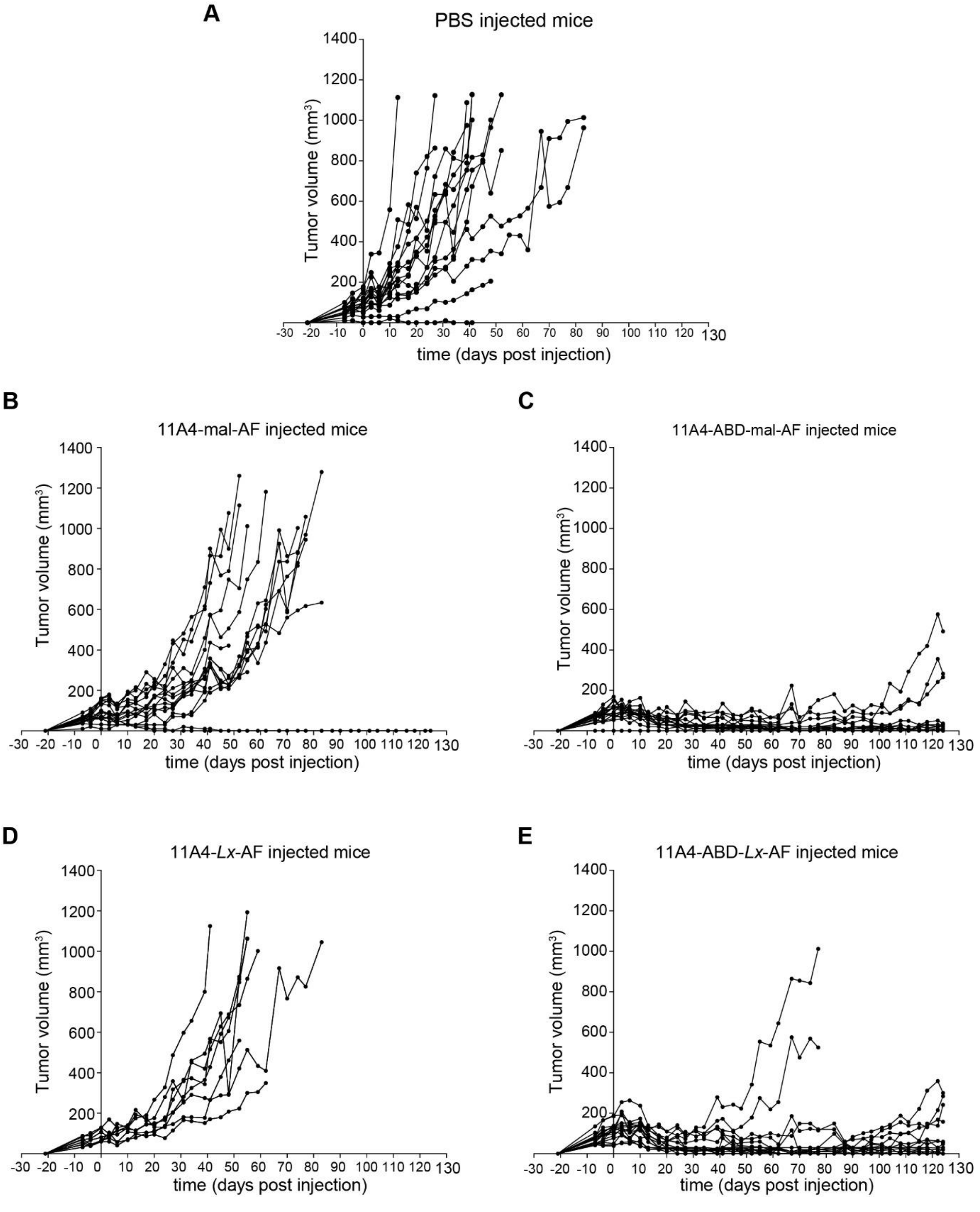

Figure S11 – Size of individual NCI-N87 tumors from mice that received a single 250 nmol/kg dose of auristatin F conjugated nanobodies: A) PBS (control), B) 11A4-mal-AF, C) 11A4-ABD-mal-AF, D) 11A4-*Lx*-AF and E) 11A4-ABD-*Lx*-AF.

Supplementary Materials

FastDigest™ enzymes, isopropyl-h-D-thiogalactopyranoside (IPTG), CaptureSelect™ C-tagXL Pre-packed Column, SnakeSkinDialysis™ Tubing (10K MWKO), Alexa Fluor™ 488 C5 Maleimide (maleimide-Alexa488), Alexa Fluor™ 647-NHS Ester (NHS-Alexa647), Zeba™ Spin Desalting Columns (7K MWCO), Nunc™ Nunclon 96-wells plates, Nunc MaxiSorp™ plates and Nunc™ Lab-TekII™ Chambered Slide™ 8-wells plates (LabTek plates) were obtained from Thermo Scientific (Thermo Fisher Scientific Inc, Breda, the Netherlands). The reagents monobasic potassium phosphate (KH_2PO_4), potassium phosphate dibasic (K_2HPO_4), magnesium chloride ($MgCl_2$), Tris base, ethylenediaminetetraacetic acid (EDTA), Tris(2-carboxyethyl)phosphine hydrochloride (TCEP in 50 mM Tris-HCl, pH 8.0), sodium borate, diethylenetriaminepentaacetic acid (DTPA), sodium chloride (NaCl), sodium phosphate (Na_3PO_4), Human Serum Albumin (HSA), Mouse Serum Albumin (MSA), HEPES, paraformaldehyde (PFA), glycine, Triton-X 100 and SIGMA*FAST*™ protease inhibitors were obtained from Sigma-Aldrich (Sigma-Aldrich Chemie NV, Zwijndrecht, the Netherlands).

Supplementary Methods

Nanobody constructs

The genes encoding for the HER2 targeted nanobody 11A4 and the irrelevant nanobody R2 were codon optimized for bacterial expression, fused to the streptococcal protein G ABD sequence, and the resulting DNA fragments were ordered from Integrated DNA Technologies (IDT BVA, Leuven, Belgium). The received ABD-containing gene constructs, as well as the 11A4 and R2 genes, were digested with SfiI- BstEII or NcoI – NotI FastDigest™ enzymes, respectively and ligated into a pelB leader sequence-containing pET28a modified vector to introduce a C-terminal free cysteine that would allow for the site-specific conjugation of a maleimide containing fluorophore or drug, followed by an EPEA affinity purification tag (GYQDYEPEA). All constructs were verified by DNA sequencing (Macrogen Europe B.V.).

Nanobody production

All nanobody constructs were expressed in BL21-CodonPlus (DE3)-RIL *E. coli* bacteria (BL21; Agilent Technologies Inc., Santa Clara, CA, USA) cultured in either shaking flasks or batch cultured in a New Brunswick™ BioFlo®/CelliGen® 115 fermentor (Eppendorf Nederland B.V.). Periplasmic nanobody expression was induced in BL21 cultures in Terrific Broth (24 g/L Yeast extract, 20g/L Tryptone, 4ml/L Glycerol, 0.017 M KH_2PO_4, 0.072 M K_2HPO_4) when OD_{600} reached 0.5-0.6 (in flask productions) or 1-2 (for fermentation) by addition of IPTG at a final concentration of 1 mM. The bacterial cultures were further incubated overnight at 25˚C with continuous shaking /agitation. Throughout fermentation, pH was 7.0 ± 0.1 (controlled by 2M NaOH and 1M H_2SO_4) and continuous agitation (maximum 1200 rpm) maintained dissolved oxygen in the medium at 70%. The following day, bacterial biomass was harvested by centrifugation at 5400g, 4ºC, and resuspended in Phosphate Buffered Saline (PBS; Lonza Benelux BV, Breda, the Netherlands). The bacterial suspension was subjected to two cycles of freezing at -20ºC and thawing followed by a centrifugation at 27000g 4ºC, resulting in the periplasmic preparation used for nanobody purification.

Nanobody purification

EPEA-tagged nanobodies were purified using either home-made anti-EPEA agarose beads or CaptureSelect™ C-tagXL Pre-packed Column attached to an ÄKTA purification system (GE Healthcare, Chicago, IL, USA), depending on the scale of protein production. Both EPEA-beads and CaptureSelect™ C-tagXL column bound nanobodies were eluted with 2 M $MgCl_2$, 20 mM Tris-HCl pH 7.0. Overnight dialysis in 10 kDa cut-off dialysis tubing against PBS followed bead-eluted nanobodies. For ÄKTA-purified constructs, a final buffer exchange step to PBS using a HiTrap™ Desalting column (GE Healthcare, Chicago, IL, USA) was included in the purification method. The purified nanobodies were analyzed with SDS-PAGE to determine the sample's purity prior to storage at -20°C until further use.

Conjugation of fluorophores

Nanobodies used in the *in vivo* imaging experiments were labeled at their C-terminal cysteine with IRDye800CW maleimide (maleimide-IR; LI-COR Biosciences, Westburg BV, Leusden, the Netherlands) or maleimide-Alexa488 following the protocol described before (1). In brief, purified protein samples were buffered exchanged to 5 mM EDTA in PBS, pH 8.0 using 7K MWCO Zeba™ columns. Disulfide bonds were reduced by adding 20 mM TCEP and room temperature (RT) incubation for 15 min. Afterwards, the buffer was exchanged to 0.4 mM EDTA in PBS, pH 7.4, maleimide-fluorophore was added to the samples (1 mg/mL) at a molar ratio of 1:4 and incubated overnight at 4°C on an overhead rotator. Finally, buffer was exchanged to PBS and free dye was removed using two consecutive Zeba™ columns.

HSA was randomly conjugated to NHS-Alexa647. For that, HSA was reconstituted in PBS at a final concentration of 1 mg/mL. The fluorescent dye was added at a 2-fold molar excess and the sample was incubated for 2 h at RT on an overhead rotator. Two consecutive Zeba™ columns were used to remove any excess of free dye.

The purity of all conjugates was evaluated with SDS-PAGE. The gel was scanned using Odyssey® Infrared Imager (LI-COR Biosciences, Westburg BV, Leusden, the Netherlands) to also determine the amount of free dye. In all cases, the amount of free dye in the final sample preparations was below 5%. The degree of conjugation (DoC) was calculated following the manufacturer's instructions using the absorbance values at 280 nm and 774 nm (IR) or 490 nm (Alexa488) or 650 nm (Alexa647), as measured by a NanoDrop™ spectrophotometer (Thermo Scientific; Thermo Fisher Scientific Inc, Breda, the Netherlands). All conjugates were stored at 4°C until further use. Probes used in *in vivo* imaging experiments were always freshly prepared before injections.

^{125}I-labeling of nanobodies

Nanobodies were labeled following the Iodogen method described by Salacinski et al (2). Samples were subjected to Size Exclusion Chromatography (SEC) and fractions of interest containing labeled probes were collected. Radioactivity of the ^{125}I-labeled nanobodies was measured using a γ-counter and the amount of free dye as well as degree of labeling and specific activity were determined.

Auristatin F nanobody drug conjugate synthesis
Free C-terminal cysteine containing 11A4 and 11A4-ABD were diluted in borate buffer (250 mM sodium borate, 250 mM NaCl and 1 mM DTPA, pH 8, in H_2O). Two molar equivalents of TCEP were added to the mixture in order to reduce the C-terminal disulphides with an incubation at 37°C for 2 h in a thermomixer (550 rpm). The reduced nanobody constructs were mixed with 10 molar equivalents of maleimide-auristatin F or AF-*Lx*-thiourea (prepared by mixing AF-*Lx*-I (3) and 20 mM thiourea (1:1) at 37°C for 2h, before addition to the reduced nanobody mixture) and incubated for 1 h on ice or at 37°C, respectively. The conjugates were purified after 4 wash cycles with PBS using 10 K MWCO Amicon® Ultra centrifugal filters (Merck Millipore, Merck KGaA, Darmstadt, Germany). Integrity of samples was evaluated with SDS-PAGE. Nanobody-AF drug conjugates were stored at -20°C until use.

NDC characterization
HPLC analysis of NDCs was performed using a Jasco HPLC system equipped with a Sepax Zenix-C SEC-300 column (300 Å, 7.8 × 300 mm) and Sepax Zenix-C SEC-300 guard column (Sepax Technologies Inc., Newark, DE, USA) using a mixture of 50 mM Na_3PO_4, 150 mM NaCl (pH 6.8), and 10 mM NaN_3, as the eluent.

LC-MS analysis was performed using a Thermo Finnigan LC system (Thermo Finnigan, San Jose, CA, USA) coupled to a Bruker Q-TOF mass spectrometer (Bremen, Germany) equipped with an electrospray ionization (ESI) source. Mass determination was performed using a Zenix-C column (4.6 x 300 mm; 5 µm; Sepax Technologies Inc., Newark, DE, USA). The mobile phase consisted of a mixture of water, acetonitrile, trifluoroacetic acid, and formic acid (79.9/19.9/0.1/0.1, v/v/v/v, respectively). A 17-minutes isocratic run was performed at a flow rate of 350 µL/minute. MS analysis was done in positive ionization mode using the following settings: ESI voltage, 4.5 kV; dry gas temperature, 190°C; dry gas flow rate, 8 L/minute; nebulizer pressure, 1.6 bar; in-source collision-induced dissociation energy, 120 eV; ion energy, 5 eV; collision cell energy, 15 eV. The protein ion charge assignment and molecular mass determinations were performed using the "Charge Deconvolution" utility of Bruker Daltonics Data Analysis software.

Determination of binding affinity on cells
To determine the nanobody binding affinity on endogenously expressed HER2 on cells, two days prior to starting the assay BT-474 and NCI-N87 cells, and the negative MDA-MB-231 cells were seeded on 96-well flat-bottomed Nunc plates at a cell density of 20000 and 8000 cells per well, respectively, and kept at 37°C, 5% CO_2. On the day of the assay, when cell confluency was around 80-90%, plates were placed at RT and further incubated for 30 min at 4°C. Cells were then washed twice with ice-cold binding buffer (1% Bovine Serum Albumin (BSA; Capricorn Scientific GmbH) and 25 mM HEPES, pH 7.2 in DMEM without phenol red (Lonza; Westburg BV, the Netherlands)). A concentration gradient of the nanobody constructs was prepared in binding buffer and added to the cells in triplicate. In the case of binding in the presence of excess serum albumin, 11A4-ABD-IR was diluted in binding medium containing two times molar excess of either HSA or MSA. Cells were subsequently incubated with the different sample concentrations for 2 h at 4°C. Signal of IR-conjugated nanobodies was immediately detected. To evaluate the binding of non-labelled nanobody constructs, cells were

further fixed following a stepwise incubation first with 2% and then 4% w/v PFA in PBS at RT for 10 min for each step. Bound nanobodies were indirectly detected after incubation with rabbit anti-VHH antibody (1:1000 in 2% w/v BSA in PBS; clone k1216, QVQ B.V.) for 1 h at RT, followed by 1 h incubation with goat anti-Rabbit IRDye800CW (1:1000 in 2% w/v BSA in PBS; LI-COR Biosciences) at RT in the dark. Wells were finally washed three times with binding buffer and 800 nm fluorescence of the bound proteins was detected using Odyssey® Infrared Imager. Fluorescent intensities were plotted (mean ± SD) over protein concentration using GraphPad Prism 8 software for Windows (GraphPad Software, San Diego, CA). Apparent binding affinity (K_D) of the different proteins was determined by non-linear regression curve fitting for one-site specific binding.

Characterization of binding on serum albumin

To determine the binding affinity of nanobody-ABD on serum albumin, 96-well flat-bottom MaxiSorp™ plates were coated overnight with 5 µg/well of either HSA, MSA or BSA in PBS at 4°C. The following day, plates were rinsed twice with PBS and blocked with 2% w/v casein/PBS for 1 h at RT. Afterwards, a decreasing concentration of 11A4-ABD-IR starting at 1 µM in 1% w/v casein, was added in the wells in triplicates. Plates were incubated at RT for 2 h, while shaking. K_Ds were determined as described above based on the fluorescent signal of the bound probes.

Evaluation of competition with serum albumin

An ELISA set up was employed to assess whether increased amount of serum albumin interferes with binding on HER2. On the day of the assay, BT-474 cells seeded on 96-well flat-bottomed plates were prepared as described above (see Binding affinity determination on cells). Then they were incubated for 2 h at 4°C with 10 nM of 11A4-ABD-IR in the presence of decreasing amounts of plasma from BALB/c mice (100% plasma; Innovative Research, Inc), in triplicate. Wells were finally washed three times with cold binding buffer and fluorescent signal at 800 nm was detected using Odyssey® Infrared Imager. Fluorescent intensities were plotted (mean ± SD) over protein concentration using GraphPad. To convert plasma dilution to molar concentration of MSA, a molecular weight of 66500 Da and a BALB/c mouse plasma concentration of 2.275 g/mL (4) were used for MSA. Non-linear regression line was fitted for one site competitive binding, with a constraint concentration of hot probe (i.e. 11A4-ABD-IR) at 10 nM and a K_D of 3.062 nM (as determined by the binding assay on BT-474 cells).

Evaluation of nanobody internalization in cells

To determine the rate at which the different constructs internalize in HER2 expressing cells, two days prior to the assay BT-474 or NCI-N87 were seeded in 96-wells plates as described above. To allow for receptor mediated internalization, cells were incubated in triplicate with a constant concentration (equal to the K_D) of IR-conjugated nanobodies at 37ºC for different times, in the presence or absence of equimolar amounts of HSA, starting from wells with the longest incubation time. At time point zero, plates were immediately transferred on ice, cells were washed with ice cold PBS and scanned at 800 nm to quantify the total fluorescence intensity. Cells were further incubated with acid wash buffer (0.2 M glycine, 150 mM NaCl, pH 2.3) for 6 min and 3 min at RT sequentially to remove the membrane bound conjugates.

Plates were again scanned at 800 nm to measure the fluorescence intensity attributed to the internalized fraction. The fluorescence of the bound fraction was inferred after subtracting the fluorescence intensity of the internalized fraction from the total fluorescent intensity. The ratio of internalized over bound fraction was plotted (mean ± SD) over time using GraphPad. A line was fitted to the data and its slope was used to determine the internalization rate constant (k_e). For representation purposes, the value of y at x=0, as calculated by the equation of the fitted line ($y= k_e \cdot x + b$), was subtracted from all time points and the resulting values were plotted over time.

In vitro cell viability assay

Cell viability of HER2 positive BT474, NCI-N87 and negative MDA-MB-231 cells treated with auristatin F nanobody drug conjugates (NDC) was evaluated by using AlamarBlue® Reagent (Bio-Rad Laboratories B.V., Veenendaal, the Netherlands). Cells were seeded in 96-wells Nunc plates (8000 cells/well for NCI-N87 and MDA-MB-231 and 10000 cells/well for BT-474) and grown for 48 h at 37°C, 5% CO_2. A concentration gradient of the NDCs, starting at 500 nM and 1:10 serial dilution steps, was prepared in cell culture medium and added to the cells in triplicate. Five days after addition of the NDCs, AlamarBlue® Reagent was directly added in the culture medium at 1:10 dilution and incubated with the cells for 2 h at 37°C. Fluorescence was measured using FluoStar Optima (Excitation filter: 550 nm and Emission filter: 590 nm; BMG LABTECH GmbH, Ortenberg, Germany). Values of the treated samples were expressed as a percentage of the control untreated cells (100% viability) and plotted over NDC concentration (mean ± SD) in GraphPad. The half maximal inhibitory concentration (IC_{50}) of the different NDCs was determined by fitting non-linear regression dose response curves with variable slopes.

Spheroid 3D cell culture and immunofluorescence staining

To obtain 3D tumor cell spheroids, BT-474 cells were cultured on top of a Matrigel® (Corning® Matrigel® Matrix; Corning BV Life Sciences, Amsterdam, the Netherlands) scaffold based on the protocol described by Lee et al (5). In brief, pre-chilled Lab-TekII™ plates were coated with a homogeneous layer of ice-cold Matrigel avoiding the formation of bubbles. The plates were then incubated for at least 30 min at 37˚C, to allow Matrigel polymerization. Single cells in suspension (8,000 cells per well) were seeded on top of the scaffold. Culture medium was refreshed every third day.

Spheroids of the desired size (<200 μm) were pre-incubated overnight at 37°C/ 5% CO_2 with 25 nM HSA-A647 in cell culture medium without phenol red. The following day, medium was replaced by a medium containing 25 nM of fluorescently labeled protein in the presence of 25 nM HSA-A647, starting with wells with the longest incubation times. Spheroids were incubated at 37°C/5% CO_2 for 15 min, 1 h, 3 h, 5 h and 24 h before the medium was removed and spheroids were rinsed twice with PBS. For fixation, samples were incubated with 2% and subsequently 4% w/v PFA for 30 min at RT. Background fluorescence, caused by any residual unreacted formaldehyde groups, was quenched by incubating the sample with 100 mM glycine (in PBS) for 30 min at RT. Samples were extensively washed three times with PBS, 10 min shaking at RT, after each step and samples were mounted with mowiol. Samples were imaged with confocal laser scanning microscope Carl Zeiss LSM700 (Carl Zeiss Microscopy GmbH,

Jena, Germany) using a 40x oil immersion objective (EC Plan-Neofluar 40x/1.30 Oil DIC, WD=0.21 mm) and 488 and 633 nm lasers. Acquisition settings were kept the same across all time points of the same probe.

Determination of percentage of radius of area covered by the fluorescent proteins
Images of the spheroids were acquired at a z-plane at the middle of the spheroid. For all different treatments and at all incubation times, a minimum of 10 spheroids were analyzed. Using ImageJ, images obtained at 488 nm (A488-labeled proteins) and 633 nm (HSA-Alexa647) were thresholded just above background levels yielding a binary image of the covered area. In these binary images, even though probe had diffused to the cell layers where clear binding is observed, the intracellular space of such cells would appear empty, accounting for non-covered area. For that, the binary image was dilated, so that intracellular space is filled. A region of interest (ROI) was drawn around the spheroid. The total surface of this ROI ($Area_{total}$) as well as the percentage accounting for the thresholded area ($Area_{thresholded}$ = x % of $Area_{total}$) were measured. Assuming each spheroid is a perfect sphere, the radius (R) was calculated as equal to the square root of the total area of each cross-section divided by π ($R = \sqrt{\frac{\mathrm{Area_{total}}}{\pi}}$). Then, the surface of the non-covered core area, which equals the total surface minus the covered surface ($Area_{not\text{-}filled} = Area_{total} - Area_{thresholded}$) and its corresponding radius ($r = \sqrt{\frac{\mathrm{Area}_{thresholded}}{\pi}}$), were also calculated. Finally, the probe's displacement (d = R-r) was expressed as a percentage of the spheroid's radius ($\frac{\mathrm{R-r}}{\mathrm{R}} \cdot 100\%$) and plotted (mean ± SD) as a function over time using GraphPad Prism.

In vivo experiments
Female athymic BALB/cOlaHsd-*Foxn1*^{Nu} (4-5 weeks old) or Nude-*Foxn1*^{Nu} mice (6-8 weeks old) mice were purchased from Envigo (Envigo, Horst, the Netherlands). All animals were housed in sterile cages under standard conditions, with food and water provided *ad libitum*. Mice intended to be used for imaging studies received chlorophyll-free diet (Envigo, Horst, the Netherlands) to eliminate food-induced fluorescence in the intestinal track. The water of mice inoculated with BT-474 cells was supplemented with 8.5 µg/mL β-estradiol (in filter-sterilized tap water). All experiments were carried out in accordance with the Dutch national law and approved by the Animal Ethical Committee board of Utrecht University (DEC no. 2014.III.10.088).

For the optical imaging study, subcutaneous tumors were induced by inoculating either 1.5×10^7 BT-474 cells (in 50% Matrigel/RPMI) or 10^6 MDA-MB-231 cells (in PBS) in both the right and left hind legs of the mice. For the efficacy study, all animals were inoculated with NCI-N87 cells by means of subcutaneous injection of 2.5×10^6 cells (in 100 µL of DMEM) in both flanks of the mouse. Tumor growth was monitored twice a week and tumor size was calculated using a digital caliper by the formula: tumor volume [mm^3] = 0.5 x length [mm] x width [mm] x width [mm].

Biodistribution

After the last imaging timepoint (72 h p.i.), mice were euthanized by cervical dislocation. Blood, tumors and several other organs were resected and weighed. Tumors were cut in two and the one half was used for the biodistribution purposes. All organs were snap frozen in liquid nitrogen immediately after resection and stored at -20°C until further processing. IR-fluorescent quantification was performed as described by (6). In brief, tissues were homogenized using TissueLyserII system (Qiagen, Venlo, the Netherlands) in RIPA buffer (50 mM Tris–HCl pH 7.4, 150 mM NaCl, 1 mM EDTA, 1% Triton X-100, 0.1% SDS) supplemented with protease inhibitors. Serial dilutions of homogenates were performed in 96-well plates alongside dilution series of a known concentration of the respective injected probe (to be used for the generation of a standard curve). Mean fluorescent intensity of the samples was measured at 800 nm using the Odyssey® Infrared Imager. The concentration of the IR-protein in the homogenates was extrapolated from the standard curve using GraphPad. The resulting values were used to calculate %ID/g. For the mice injected with ^{125}I-nanobodies, the collected organs were weighed, and radioactivity was directly counted with a γ-counter. The resulting values were used to calculate %ID/g.

Fluorescence imaging and immunohistochemical analysis of resected tumors

The second half of each tumor, collected after animals were euthanized, was used for histological analysis. Immediately after resection, the tumor pieces were fixed in neutral buffered formalin and routinely processed in paraffin blocks that were stored in the dark until further processing. Sections of 4 μm thickness were first scanned at 800 nm using the Odyssey® Infrared Imager. Afterwards, immunohistochemistry for HER2 detection (Rabbit anti-c-erbB-2, clone SP3, Thermo Fisher Scientific Inc) and hematoxylin and eosin (H&E) staining were performed as described before (7).

Supplementary references

1. Kijanka M, Warnders FJ, El Khattabi M, Lub-De Hooge M, Van Dam GM, Ntziachristos V, et al. Rapid optical imaging of human breast tumour xenografts using anti-HER2 VHHs site-directly conjugated to IRDye 800CW for image-guided surgery. Eur J Nucl Med Mol Imaging. 2013;40:1718–29.
2. Salacinski PRP, McLean C, Sykes JEC, Clement-Jones V V., Lowry PJ. Iodination of proteins, glycoproteins, and peptides using a solid-phase oxidizing agent, 1,3,4,6-tetrachloro-3α,6α-diphenyl glycoluril (Iodogen). Anal Biochem. 1981;
3. Merkul E, Sijbrandi NJ, Aydin I, Muns JA, Peters RJRW, Laarhoven P, et al. A successful search for new, efficient, and silver-free manufacturing processes for key platinum(ii) intermediates applied in antibody-drug conjugate (ADC) production. Green Chem. 2020;
4. Zaias J, Mineau M, Cray C, Yoon D, Altman NH. Reference values for serum proteins of common laboratory rodent strains. J Am Assoc Lab Anim Sci. 2009;
5. Lee GY, Kenny PA, Lee EH, Bissell MJ. Three-dimensional culture models of normal and malignant breast epithelial cells. Nat Methods. 2007;
6. Oliveira S, Cohen R, van Walsum MS, van Dongen GAMS, Elias SG, van Diest PJ, et al. A novel method to quantify IRDye800CW fluorescent antibody probes ex vivo in tissue distribution studies. EJNMMI Res. 2012;

7. Vermeulen JF, van Brussel ASA, van der Groep P, Morsink FHM, Bult P, van der Wall E, et al. Immunophenotyping invasive breast cancer: Paving the road for molecular imaging. BMC Cancer. 2012;
